# Mediated Plastid RNA Editing in Plant Immunity

**DOI:** 10.1371/journal.ppat.1003713

**Published:** 2013-10-31

**Authors:** Javier García-Andrade, Vicente Ramírez, Ana López, Pablo Vera

**Affiliations:** Instituto de Biología Molecular y Celular de Plantas, Universidad Politécnica de Valencia-C.S.I.C, Ciudad Politécnica de la Innovación, Ingeniero Fausto Elio, Valencia, Spain; Ohio State University, United States of America

## Abstract

Plant regulatory circuits coordinating nuclear and plastid gene expression have evolved in response to external stimuli. RNA editing is one of such control mechanisms. We determined the Arabidopsis nuclear-encoded homeodomain-containing protein OCP3 is incorporated into the chloroplast, and contributes to control over the extent of *ndhB* transcript editing. *ndhB* encodes the B subunit of the chloroplast NADH dehydrogenase-like complex (NDH) involved in cyclic electron flow (CEF) around photosystem I. In *ocp3* mutant strains, *ndhB* editing efficiency decays, CEF is impaired and disease resistance to fungal pathogens substantially enhanced, a process recapitulated in plants defective in editing plastid RNAs encoding NDH complex subunits due to mutations in previously described nuclear-encoded pentatricopeptide-related proteins (i.e. CRR21, CRR2). Furthermore, we observed that following a pathogenic challenge, wild type plants respond with editing inhibition of *ndhB* transcript. In parallel, rapid destabilization of the plastidial NDH complex is also observed in the plant following perception of a pathogenic cue. Therefore, NDH complex activity and plant immunity appear as interlinked processes.

## Author Summary

Plastids originated from cyanobacteria that were incorporated into the eukaryotic cell through an endosymbiotic relationship. During the gradual evolution from endosymbiont to organelle, most genes of the cyanobacterial genome were transferred to the nuclear genome. Therefore, plastid biogenesis and function relies on nuclear gene expression and the import of these gene products into plastids, with the molecular dialogue between these two plant cell compartments therefore needing a precise coordination. Nuclei-to-chloroplast communication, and *vice versa*, are thus regulated through anterograde and retrograde signaling pathways, respectively. Post-transcriptional RNA editing of plastid RNAs by nuclear encoded regulatory proteins, such as pentatricopetide repeat (PPRs) proteins, represents one of such mechanisms of control. Through the characterization of the nuclear-encoded OCP3 protein, previously found to function as a disease resistance regulator in Arabidopsis, we have discovered a pathogen-sensitive editing-mediated control of the plastidial NDH complex involved in cyclic electron flow (CEF) around photosystem I. This led us to find that different PPRs controlling editing extent of transcripts for plastidial NDH complex are modulated by pathogenic cues. Our results thus represent the first series of evidence indicating engagement of chloroplast RNA editing and chloroplast NDH activity in plant immunity.

## Introduction

Plastid function relies on nuclear gene expression, and the import of nuclear gene products into plastids [1]. In fact, the plastid genome of current land plants encodes 75–80 proteins [2], whereas nuclear-encoded chloroplast proteins are estimated between 3500 and 4000 [3]. Current data approximates several hundred nuclear-encoded proteins are involved in post-transcriptional regulation of plastid gene expression [4], [5]. One such regulation is mediated through RNA editing, a post-transcriptional process that alters specific cytidine residues to uridine (C-to-U) in different plastid RNAs [6]. Thirty-four sites are edited in 18 transcripts of Arabidopsis plastids [7]. Among the nuclear-encoded proteins regulating RNA editing, the pentatricopeptide repeat (PPR) protein family has attracted notable interest [8]. This family comprises 450 members defined by a tandem array of PPR motifs. PPRs are also involved in almost all stages of plastid gene expression, including splicing, RNA cleavage, translation, and RNA stabilization [9]. The pioneer work of Kotera *et al*. [10], [11] revealed the Arabidopsis PPR protein CHLORORESPIRATORY REDUCTION4 (CRR4) acts as a site-specific recognition factor for RNA editing of the site 1 (ndhD-1) in the plastid *ndhD* transcript. *ndhD* encodes the D subunit of the chloroplast NADH dehydrogenase-like complex (NDH), involved in cyclic electron flow (CEF) around photosystem I (PSI) [11], [12]. Consequently, *crr4* mutants are defective in *ndhD* transcript editing at the ndhD-1 site, and CEF is compromised [10], [11]. Subsequently, the number of PPR-encoding genes participating in editing control in the chloroplast has enlarged [9]. Although empirical evidence has been demonstrated for only a few PPR proteins, it is currently accepted that PPR proteins act as sequence-specific RNA binding adaptors, and hypothetical inferences suggest PPRs recruit effector enzymes or proteins to the target RNAs [13], [14]. While the mechanism by which specific PPR proteins recognize specific editing sites is becoming understood, questions still remain to be completely solved including the characterization of the molecular components that conform the RNA editing apparatus (editosome) or the still unsolved identification of editing enzyme itself. Therefore, identification of additional components modulating editing activities in plastids, and ascertaining how control of the post-transcriptional mechanism of chloroplast function influences other biological processes, in particular immune responses, is of great importance.

Despite the critical role of chloroplasts as a site for production of integral mediators of plant immunity such as salicylic acid, jasmonic acid, and ABA [15], the molecular link between chloroplasts and the nuclear-encoded immune system remains largely unexplored. MEcPP, a plastidial metabolite previously shown to be involved in activating plant immunity in Arabidopsis [16] has been shown to mediate a retrograde signaling regulating expression of nuclear stress-response genes [17]. Nomura *et al*. [18] recently reported the chloroplast calcium-sensing receptor (CAS), involved in transducing changes in cytosol Ca^2+^ concentrations into chloroplast responses, regulates plant immunity in Arabidopsis, possibly through chloroplast-derived ROS signals (i.e. ^1^O_2_ and H_2_O_2_; [19]). These ROS signals may function through a retrograde signaling pathway to activate the expression of nuclear genes. Terashima *et al*. [20] demonstrated CAS is a crucial component of the machinery driving CEF around photosystem I in *Chlamydomonas reinhardtii*, and suggested CAS mediates changes in CEF activity, however the mechanism remains unresolved. In contrast to linear photosynthetic electron flow, where light drives ATP and NADPH synthesis; during CEF, light only drives ATP production by cycling electrons around PSI and Cyt *b*6*f* complexes, providing the molecular basis for this major energetic switch. Concurrently, CEF leads to the reduction of the plastoquinone pool, thereby increasing the frequency of charge recombination events in PSII; and as a result, altering the chloroplast redox status [21]. Consequently, CAS and Ca^2+^ via CEF alter ROS homeostasis, and may activate ROS-mediated retrograde signaling, which in a plant-pathogen interaction may have an impact on the outcome of plant disease resistance. CEF is also interrelated with nonphotochemical quenching (NPQ) which protects plants against damage resulting from ROS formation [22]. Göhre *et al*. [23] recently reported defense activation during PAMP-triggered immunity (PTI) in Arabidopsis resulted in rapid NPQ decrease, and NPQ also influenced immune responses, suggesting that NPQ and CEF are integral components regulating plant defense response. Similarly, chloroplast-generated ROS following the recognition of pathogen-derived effectors by plant R proteins, resulted in HR-type programmed cell death (PCD), demonstrating chloroplast contribution to effector-triggered immunity (ETI) [24]. These evidences emphasize the importance of chloroplasts in plant immunity, and indicate the potential for future discoveries in this area of research.

We show the disease resistance regulator OVEREXPRESSOR OF CATIONIC PEROXIDASE3 (OCP3) is targeted to the chloroplast, and controls editing efficiency of plastid *ndhB* transcripts. We also show that NDH activity, and therefore CEF around PSI, is an important control point in plant immunity. Furthermore, a previously undescribed signaling pathway linking editing control with plant immunity via CEF activity modulation in the chloroplast was elucidated in this study.

## Results

### OCP3 is targeted to plastids

**Figure 1:**
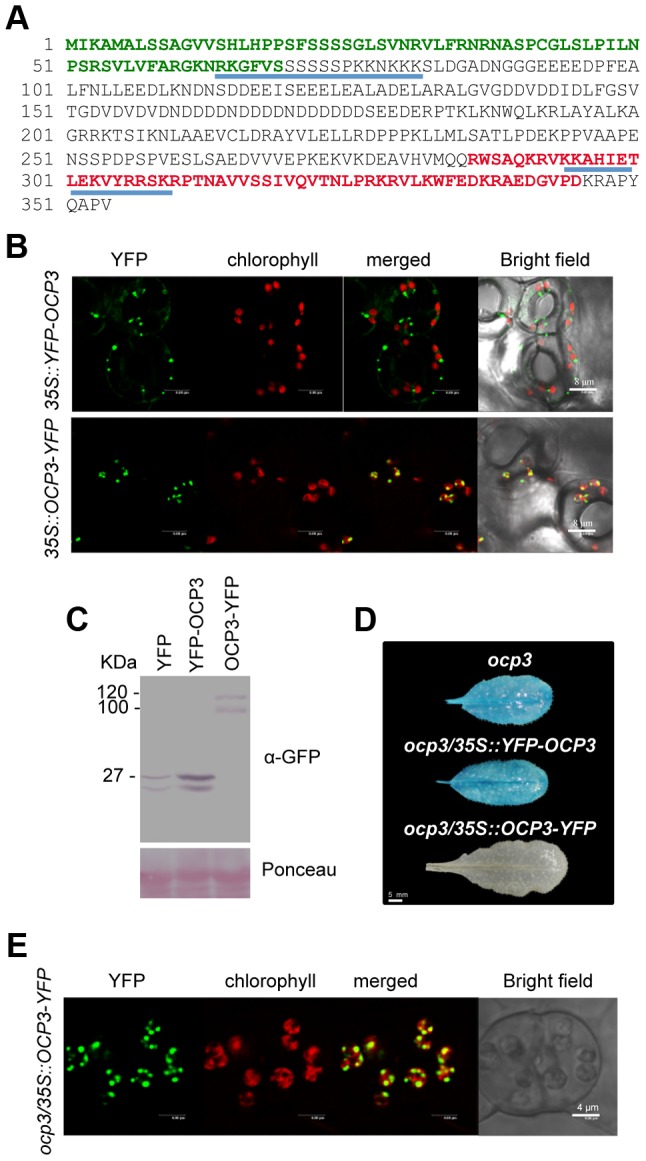
Functional OCP3 resides in the chloroplast. (**A**) OCP3 amino acid sequence. The 60 amino acid residues conforming the homeodomain are indicated in red letters. The N-terminal signal peptide sequence to chloroplast targeting, as predicted by TargetP, is indicated in green letters. Two canonical bipartite nuclear localization signals (RK-(X)10-KKNKKK and KK-(X)10-RRSKR) are underlined in blue. (**B**) Fluorescent confocal microscopy evaluation of protein localization in transgenic Arabidopsis plants transformed with a *35S::YFP-OCP3* construct (upper panel) and a *35S::OCP3-YPF* constructs (lower panel). YFP-specific fluorescence is shown in green and chlorophyll-derived fluorescence is shown in red. (**C**) Western blot analysis using anti-GFP antibodies of crude protein extracts derived from Arabidopsis plants transformed with *35S::YFP*, *35S::YFP-OCP3* and *35S::OCP3-YPF* genes constructs, respectively. Molecular mass markers are shown on the left. (**D**) Characteristic GUS expression pattern, as driven by the *Ep5C* gene promoter, in leaves of the *ocp3* mutant. Complementation of this molecular phenotype in *ocp3* plants upon transformation with *35S::OCP3-YFP* but not upon transformation with *35S::YFP-OCP3*. (**E**) Fluorescent confocal microscopy evaluation of protein localization in transgenic *ocp3* plants transformed with a *35S::OCP3-YPF* construct.

OCP3 was classified as a transcription factor as it contained a 60-amino acid domain resembling a homeodomain and carried also two canonical bipartite nuclear localization signals [25] (Figure 1A). These features were interpreted as indicative of targeting OCP3 to nuclei, where it would be functioning as a negative regulator of plant immunity and was congruent with *ocp3* plants exhibiting a remarkable enhanced resistant to fungal pathogens due to a primed immune state [25]–[27]. However, when *Arabidopsis* were transformed with a gene construct carrying the fluorescent YFP protein fused to the OCP3 N-terminus or, alternatively, to the C-terminus (i.e. *35S::YFP-OCP3* and *35S::OCP3-YPF* constructs, respectively), confocal microscopy revealed different subcellular localizations for each protein (Figure 1B). YFP-OCP3 expression led to YFP-specific fluorescence at dispersed positions within the cell, while that derived from OCP3-YFP was unequivocally localized to the chloroplast (Figure 1B). These different protein localizations were reproduced upon transfecting tobacco protoplasts using the same constructs (Supplemental Figure S1). Western blot using an anti-GFP antibody, revealed YFP-OCP3 accumulated as a YFP immunoreactive band similar to that observed for free YFP (Figure 1C). This was interpreted as partial trimming or processing of YFP-OCP3. Conversely, the OCP3-YFP protein was stable, and accumulated as two low migrating immunoreactive bands; the molecular weight of these polypeptides congruent with that expected for a fusion OCP3 with YFP (Figure 1C). Furthermore, the *35S::OCP3-YFP* gene construct, but not *35S::YFP-OCP3*, complemented the *ocp3* phenotype following stable transformation (Figure 1D). The *ocp3* mutant line carried a copy of the pathogen- and H_2_O_2_-responsive *Ep5C::GUS* transgene that became constitutively active in the mutant (revealed after staining with X-gluc) [25], [28], therefore, complementation was recorded as *Ep5C::GUS* expression repression (Figure 1D). Eight independent *ocp3/35S::OCP3-YFP* lines were assayed, and all showed repressed GUS expression (Figure 1D), indicative of effective complementation; in all cases the OCP3-YFP protein was targeted to the chloroplast (Figure 1E). However, all 12 independent *ocp3/35S::YFP-OCP3* transformed lines we generated retained GUS expression driven by the *Ep5C* gene promoter, demonstrating the inability of YFP-OCP3 to complement *ocp3*.

Inspection of OCP3 with TargetP (http://www.cbs.dtu.dk/services/ChloroP/) revealed a predicted 69 amino acid chloroplast signal peptide (SP) (Figure 1A). Correspondingly, N-terminal amino acid sequencing using Edman degradation of OCP3-YFP protein revealed processing at the predicted site (Polyp2; Supplemental Figure S2A). Furthermore, fusion of the first 81 amino acids of OCP3 to YFP (i.e. OCP3_1–81_-YFP) was sufficient to target and internalize YFP to the chloroplast (Supplemental Figure S2B–C). Interestingly, chloroplast targeting, but not internalization, occurred when a short deletion (from aa 68-to-74) was introduced in the constructs (OCP3Δ_68–74_-YFP; Supplemental Figure S2B–C), indicating amino acids at position 68-to-74 were critical for proteolytic maturation of OCP3 in the chloroplast. These results were congruent with the absence of *ocp3* complementation with deleted OCP3Δ_68–74_-YFP, and lack of mature OCP3-YFP protein accumulation in transformed plants (Supplemental Figure S2D-E). Immunoblot analysis of chloroplast suborganellar fractionations derived from plants expressing OCP3-YFP revealed incorporation of the protein into the chloroplast, and enrichment in the stroma and thylakoid fractions (Supplemental Figure S2F). Collectively, these results indicated a functional OCP3 protein resides in the chloroplast.

### OCP3 localization overlaps with pTAC2, a pentatricopeptide repeat (PPR) protein

**Figure 2:**
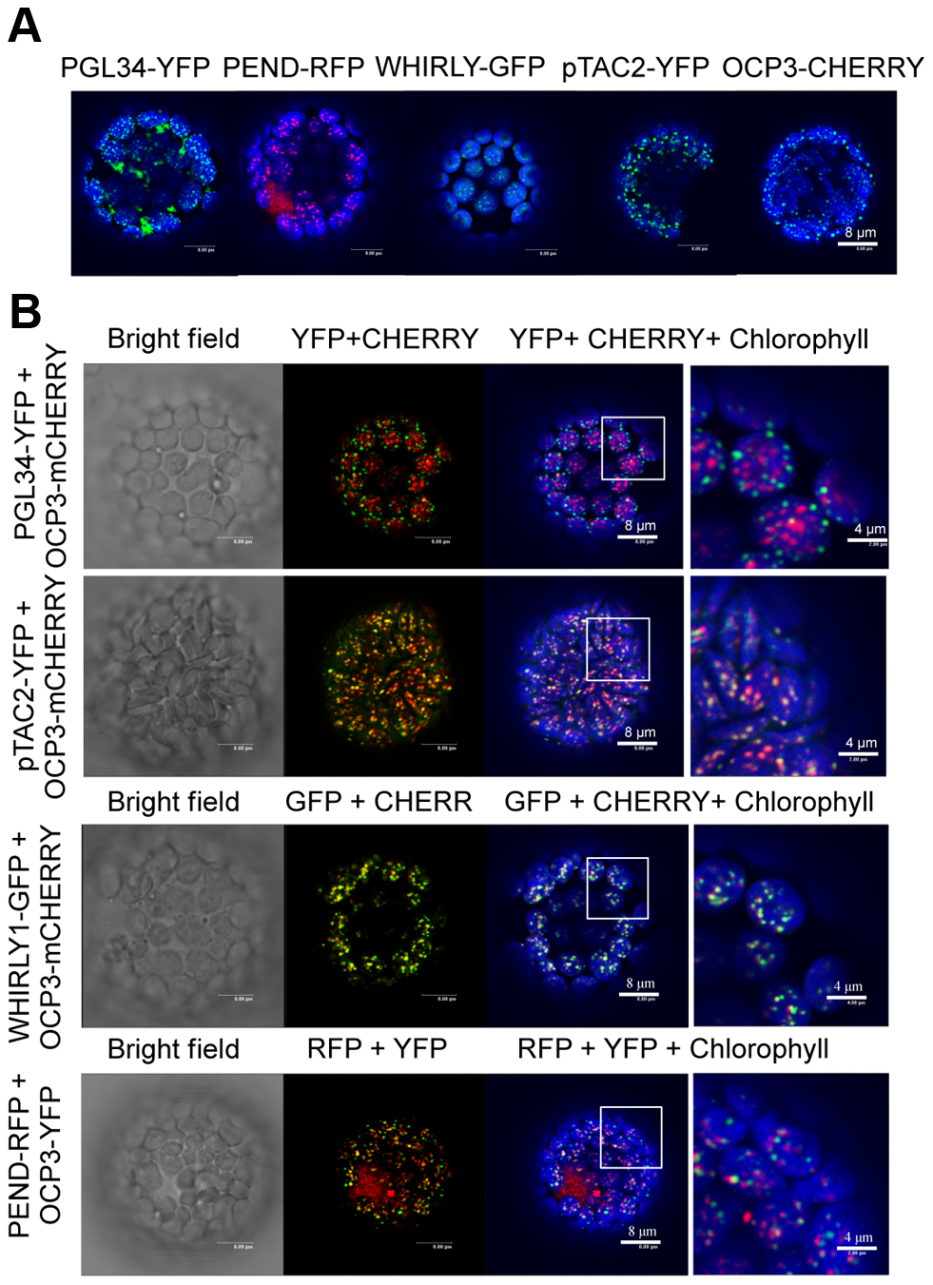
OCP3 co-localizes with pTAC2. (**A**) Chloroplast localization pattern of PGL34-YFP, PEND-RFP, WHIRLY-GFP, pTAC2-YFP and OCP3-mCHERRY in protoplasts from *N. benthamiana* evaluated by confocal microscopy. (**B**) Co-localization patterns of OCP3 with each of the proteins shown in (A). YFP- and GFP-specific fluorescence is shown in green, RFP- and mCHERRY-specific fluorescence is shown in red and chlorophyll-derived fluorescence is shown in blue.

OCP3-YFP distribution within the chloroplast showed protein accumulated in the form of speckles or punctate patterns. We noted similarities among proteins targeting different plastid structures or molecules, including plastoglobules (i.e. PGL34, [29]), plastid nucleoids (PEND, [30]), targeting associated with introns containing RNAs (i.e. WHIRLY, [31]) and with RNAs undergoing editing (i.e. pTAC2) (Figure 2A). We co-expressed the OCP3-mCherry protein with PGL34-YFP, PEND-RFP, WHIRLY-GFP, or pTAC2-YFP in protoplasts to demonstrate possible co-localizations. OCP3-mCherry fluorescence overlapped consistently with the fluorescence derived from the pentatricopeptide repeat (PPR) protein pTAC2-YFP (Figure 2B).

### *OCP3* is co-expressed with a cluster of nuclear genes encoding plastid PPR proteins

**Figure 3:**
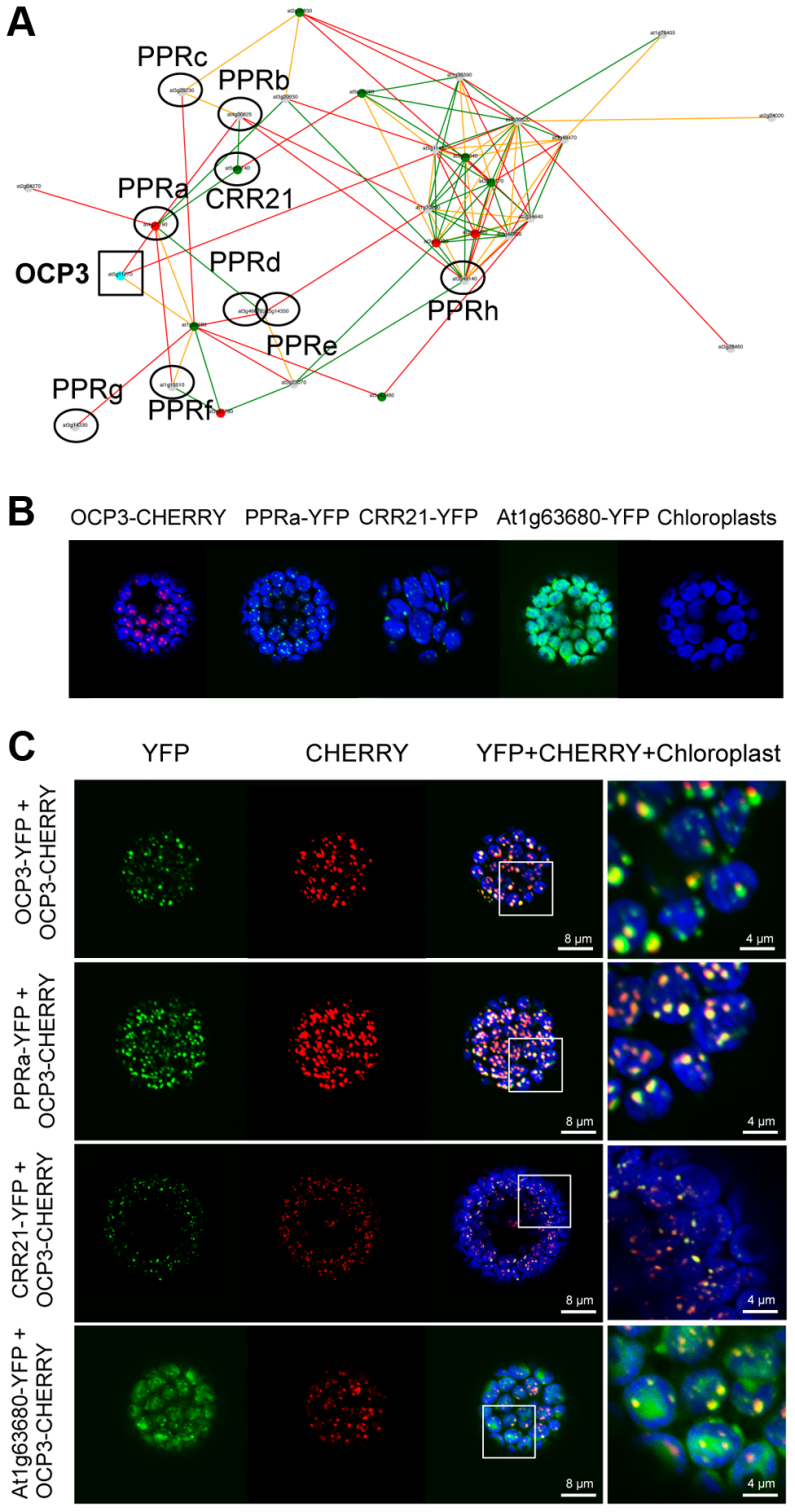
OCP3 is co-regulated with a subset of nuclear-encoded chloroplast PPR proteins. (**A**) Co-expression gene vicinity network around the *OCP3* node. This gene cluster was partitioned from the complex network illustrated in Supplementary Figure S3 and shows co-expressed genes that are only two steps away from *OCP3*. Nine out of the 31 genes of the cluster (see Table S2) encode PPR proteins and are indicated by circles, while *OCP3* is indicated by a square. In addition to *CRR21* (at5g55740), the other 8 *PPRs* surrounding *OCP3* node have been tentatively name as *PPRa* (at4g21190), *PPRb* (at4g30825), *PPRc* (at3g29230), *PPRd* (at3g46610), *PPRe* (at5g14350), *PPRf* (at1g15510), *PPRg* (at3g14330) and *PPRh* (at3g49140). Nodes indicate individual genes, and edges indicate whether two genes are co-expressed above a certain mutual rank. Color codes for nodes and edges as in Supplementary Figure S3. Green, orange and red edges indicate a mutual rank relationship 10 (green), 10 but 20 (orange) and 20 but 30 (red), respectively, for each connected gene pair. GO characterization of the 31 genes is shown in Table 2. The network was generated, and modified from AraGenNet (http://aranet.mpimp-golm.mpg.de/aranet). (**B**) Chloroplast localization pattern of OCP3-CHERRY, PPRa (at4g21190)-YFP, CRR21-GFP, and At1g63680-YFP in protoplasts from *N. benthamiana* transfected with each respective construct as evaluated by confocal microscopy. (**C**) Chloroplast co-localization patterns of OCP3-CHERRY with OCP3-YFP and of OCP3-CHERRY with each of the other proteins shown in (B) in protoplast co-transfected with each of the indicated construct pairs. YFP-specific fluorescence is shown in green, CHERRY-specific fluorescence is shown in red and chlorophyll-derived fluorescence is shown in blue.

Transcriptionally coordinated genes tend to be functionally related [32]. We hypothesized that identification of genes that are co-expressed with *OCP3* (at5g11270) in Arabidopsis would provide clues into the biological processes of OCP3 in the chloroplast. Therefore, we initially identified a co-expressed gene vicinity network for *OCP3* using the AraGenNet platform (http://aranet.mpimp-golm.mpg.de/) [33] where *OCP3* matched cluster 49 (Supplemental Figure S3). Based on functional annotation using MapMan ontology terms (http://aranet.mpimp-golm.mpg.de/), the co-expression network contained 207 genes significantly enriched for biochemical and regulatory aspects related to chloroplast development and function (see Table S1). A closer vicinity network including only genes two steps away from *OCP3*, identified 31 genes that were all related to plastid processes (Figure 3A and Table S2). Nine of these genes encoded PPR proteins, and the biological role in only one of these *PPR* genes, *CRR21* (at5g55740), has been elucidated. CRR21 acts as a site-specific factor recognizing RNA editing site 2 (ndhD-2 site) in plastid *ndhD* transcript, suggesting that the Ser128Leu change has important consequences for the function of the NDH complex [13]. The remaining eight *PPR* genes, were tentatively named as follows: *PPRa* (at4g21190), *PPRb* (at4g30825), *PPRc* (at3g29230), *PPRd* (at3g46610), *PPRe* (at5g14350), *PPRf* (at1g15510), *PPRg* (at3g14330), and *PPRh* (at3g49140) (Figure 3A).

As for pTAC2, plastidial overlapping localization pattern was observed for OCP3-mCHERRY and PPRa-YFP (Figure 3B–C). Similarly, OCP3-mCHERRY overlaps with CRR21-YFP both following the same punctate distribution pattern (Figure 3B–C). The common co-localization pattern of OCP3 with different PPR proteins was not followed by all other proteins whose genes where co-expressed with OCP3, as deduced from the non-overlapping localization pattern in OCP3-mCHERRY and AT1G63680-YFP (Figure 3B). The observed common localization of OCP3 and different PPR proteins suggested involvement of OCP3 in some aspects of plastidial RNA editing processes

### OCP3 is required for RNA editing of *ndhB* transcript in plastids

**Figure 4:**
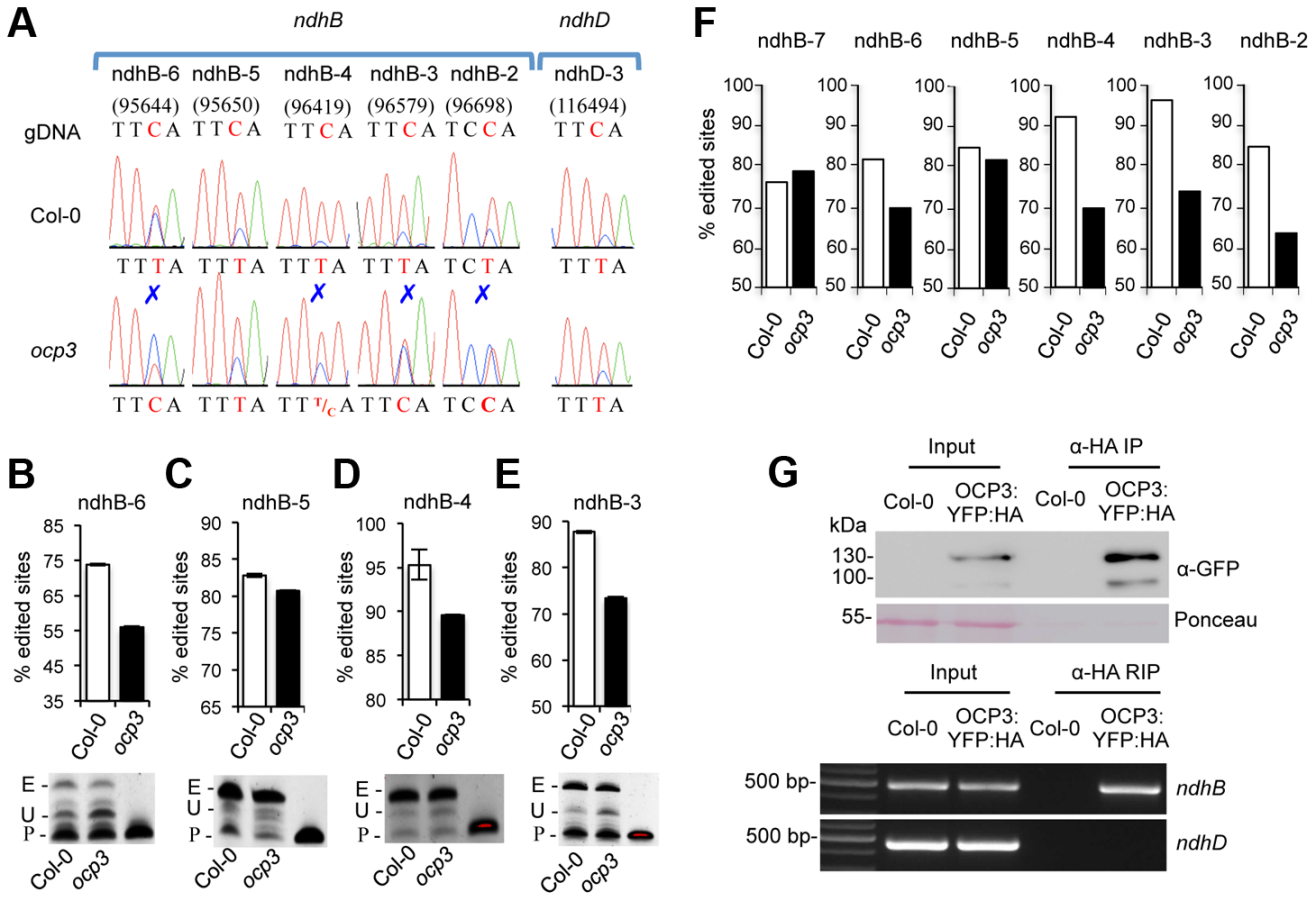
Editing defects in *ocp3* plants and *in vivo* association of OCP3 with *ndhB* RNA. (**A**) Nucleotide sequences surrounding the RNA editing sites of ndhB-6 (95644), ndhB-5 (95650), ndhB-4 (96419), ndhB-3 (96579), ndhB-2 (96698) and ndhD-2 (116494) are shown as sequence chromatograms. The editing sites are specified relative to the nucleotide sequence of the complete Arabidopsis chloroplast genome (Genebank accession number AP000423). Editing sites are indicated by a red C residue in the genomic (gDNA) sequence and its conversion or not to a U(T) residue in Col-0 and *ocp3* derived RNA samples. Editing defects in *ocp3* are indicated by a blue mark above the corresponding peaks. (**B–E**) Poisoned primer extension (PPE) assays were conducted on the editing sites ndhB-6 (B), ndhB-5 (C), ndhB-4 (D) and ndhB-3 (E). RT-PCR products were obtained with labeled 6-carboxyfluorescein primers that anneals next to the target editing site (forward PPE primers were used for all sites). Acrylamide gels (below panels) were visualized under UV light, and intensity of bands quantified calculated and plotted. Bars represent mean ± SD, n = 3 independent replicates. Experiments were repeated at least three times with similar results. E, edited; U, unedited, P; primer. (**F**) Comparative RNA editing efficiency in Col-0 and *ocp3* plants as quantified from direct DNA sequencing of 100 independent cDNAs per genotype encompassing each of the indicated editing sites. (**G**) RNA immunoprecipitation (RIP) of anti-HA precipitated protein complexes from leaves derived from Col-0 and a 35S::OCP3:YFP:HA transgenic line. The upper panel shows a Western blot of protein present in crude leaf extracts and proteins immunoprecipitated (IP) with anti-HA antibody. The blot was developed with anti-GFP antibody and shows enrichment of the OCP3:YFP:HA protein in samples derived from the transgenic line. In the lower panel RT-PCR was used to detect association of *ndhB* transcripts with OCP3-enriched complexes in comparison to the corresponding input sample. Lack of association of *ndhD* transcripts with OCP3-enriched complexes is shown as a negative control.

To test the involvement of OCP3 in RNA editing, we systematically examined the editing status of chloroplast transcripts derived from wild type and *ocp3* plants using high-resolution melting (HRM) screen of the 34 sites undergoing editing in Arabidopsis [7]. We identified major defects in the RNA editing of *ndhB-6*, *nhdB-4*, *ndhB-3*, and *ndhB-2* sites in *ocp3* plants (Figure S4). The comparison of the sequencing electrophoregrams of the RT-PCR products surrounding the editing sites confirmed that editing was compromised at the four indicated sites, if not totally at least partially, in *ocp3* plants (Figure 4A and Figure S6). All other known sites appeared similarly edited in *ocp3* plants as in Col-0 plants. Editing defects were further confirmed by poisoned primer extension (PPE) assays (Figure 4B–E). ndhB-6, ndhB-4, and ndhB-3 sites were edited in Col-0 at estimated efficiencies of approximately 72%, 95%, and 88%, respectively, while in *ocp3* plants efficiencies were reduced approximately to 55%, 89%, and 72%, respectively (Figure 4B and 4D). The ndhB-2 editing site possessed a contiguous cytosine residue adjacent to the cytosine to be edited, which impeded a reliable PPE assay. Therefore, ndhB-2 was not further studied by this method. The ndhB-5 site exhibited no editing variation between Col-0 and *ocp3*, with efficiencies in the range of 82.5% and 81%, respectively; therefore, it served as an internal editing control site for the *ndhB* transcript not affected in *ocp3* plants.

Sequencing of individual cDNA clones, in sufficient quantities, is considered the most accurate method to measure editing extent, but is not cost effective for large-scale studies. We sequenced individual cDNA clones derived from RNAs obtained from equivalent leaves from Col-0 and *ocp3* plants. cDNA cloning strategy was designed to include the ndhB-4, ndhB-3, and ndhB-2 sites in one amplicon (amplicon I) and the ndhB-6 site, along with non-altered ndhB-5, and ndhB-7 sites, in another amplicon (amplicon II). One hundred cDNA clones for each amplicon, and for each genotype were analyzed by direct sequencing. Editing efficiency comparison is shown in Figure 4F. For the ndhB-4 site, Col-0 showed a 92% (92 of 100 sequenced clones) editing extent, which was reduced to 71% in *ocp3* plants. Col-0 showed a 94% editing extent for ndhB-3, reduced to 77% in *ocp3*. The ndhB-2 site exhibited an editing extent of 86% in Col-0, which was reduced to 64% in *ocp3*. These values were comparable to those observed for the PPE assays. Interestingly, *ocp3* plants exhibited concurrent editing inhibition at two sites within the same cDNA clone (of the three potential ones in amplicon I) in 21% of the sequenced clones while in Col-0 it was only 3%. Furthermore, lack of concurrent editing at the three sites remained notable in *ocp3*, and was observed in 8% of the sequence clones while in Col-0 it was 0%. These results suggested the concomitant editing inhibition at more than one site on the same *ndhB* transcript was a common feature in editing defects in *ocp3* plants. The ndhB-6 site showed an editing extent of 81% in Col-0 plants, which was reduced to a 61% in *ocp3* plants (Figure 4F). ndhB-5 and ndhB-7 served as controls for non-variation sites within the same transcript, and the editing extent was similar between Col-0 and *ocp3* plants (83% reduced to 81% for ndhB-5; and 76% increased to 78% for ndhB-7) (Figure 4F). Collectively these data indicated that OCP3 is required for efficient *ndhB* transcript edition.

### *In vivo* association of OCP3 with *ndhB* RNA

To directly assess the association of OCP3 with *ndhB* transcripts, leaves from Col-0 and from a transgenic line expressing a *35S::OCP3:GFP:HA* gene construct were treated with folmaldehyde to generate protein-RNA cross-links and subsequently subjected to RNA immunoprecipitation (RIP), an analysis that serves to detect the presence of the corresponding RNA in the protein immunoprecipitate by reverse transcription PCR (RT-PCR). Immunoprecipitation of crude protein extracts with an anti-HA antibody selectively enriched the chimeric OCP3 protein in samples derived from the 35S::OCP3:YFP:HA overexpressing line (Figure 4G, upper panel). Interestingly, the immunoprecipitated OCP3 complexes were shown to specifically co-precipitate *ndhB* transcripts as revealed by comparative RT-PCR analysis of the corresponding samples derived from the transgenic line and Col-0 plants (Figure 4G, lower panel). *ndhD* transcripts, which served as a negative control, did not show association with the OCP3 complex (Figure 4G, lower panel). The results thus indicate that OCP3 associates *in vivo* with *ndhB* transcript. Whether this association is the result of a direct interaction of OCP3 with the RNA molecule, or rather a consequence of the interaction of OCP3 with an RNA binding protein recognizing specifically RNA sequences of the *ndhB* transcript remains unknown. Future characterization of such protein complex and the elucidation of its associated biochemical function will shed light on how editing of the *ndhB* RNA at their multiple editing sites is regulated.

### *ocp3* plants are impaired in NDH activity

**Figure 5:**
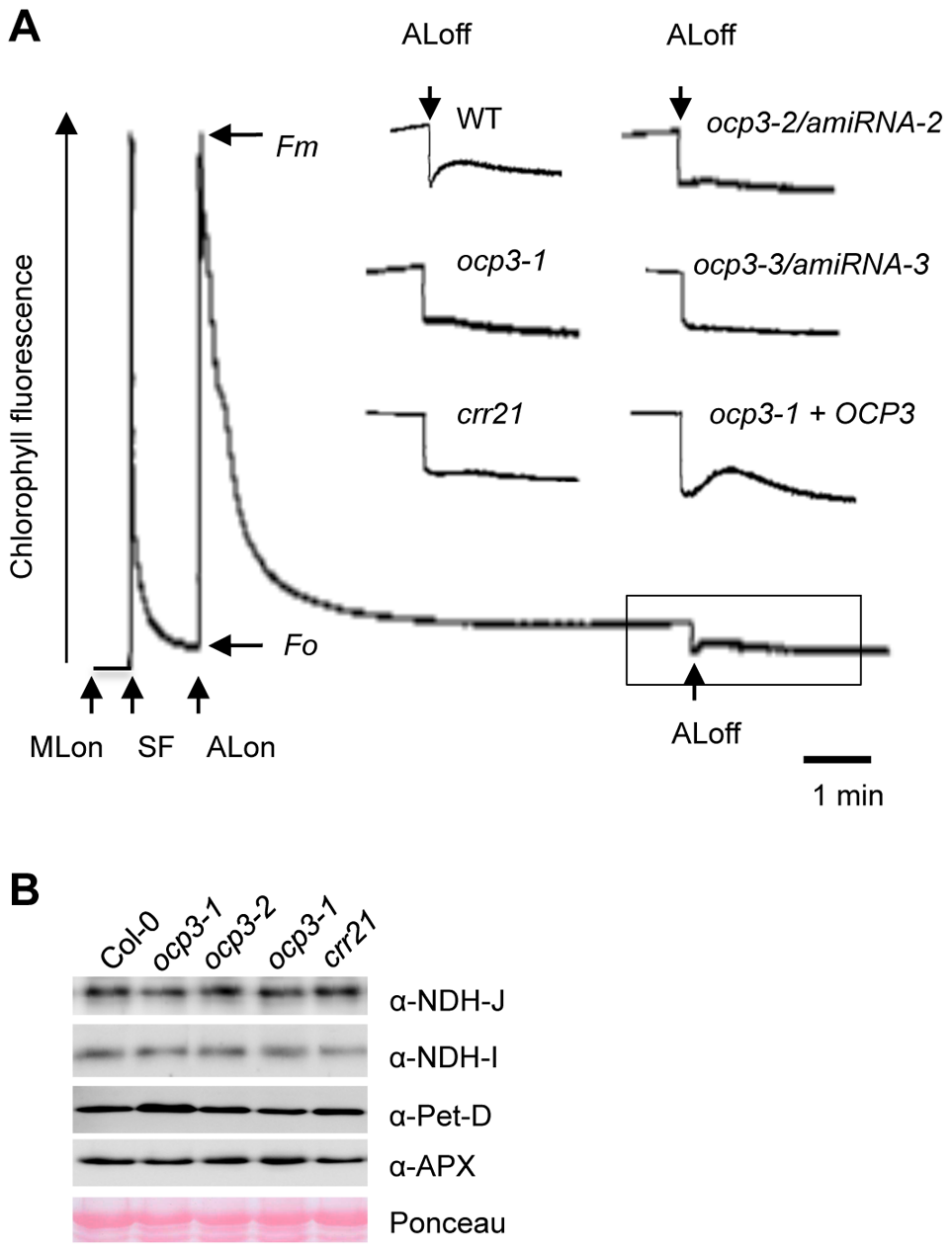
Monitoring of NDH activity by using chlorophyll fluorescence analysis. (**A**) Analysis of the transient increase in chlorophyll fluorescence (apparent Fo) after termination of actinic light (AL) illumination. The bottom curve indicates a typical trace of chlorophyll fluorescence in the wild type (WT). Leaves were exposed to AL (50 µmol photons m^−2^ s^−1^) for 5 min. AL was turned off and the subsequent transient rise in fluorescence caused by plastoquinone reduction based on NDH activity monitored. Insets are magnified traces from the boxed area. The fluorescence levels were normalized by the maximum fluorescence at close PSII centers in the dark (*Fm*) levels; ML, measuring light; SF, a saturating flash of white light. The fluorescence was monitored using a pulse-amplitude-modulation chlrophyll fluorometer. *ocp3-1*+*OCP3*, *ocp3-1* allele transformed with a wild type *OCP3* cDNA. (**B**) Immunoblot analysis of NDH NdhI and NdhJ subunits, the subunit IV of cytochrome *b6f* (Pet-D) and ascorbate peroxidase (APX). Proteins were extracted from chloroplast preparations from each genotype and lanes loaded with 20 µg protein.

Normal RNA editing at *ndhB-6*, *ndhB-4*, *ndhB-3* and *ndhB-2* sites converts a Ser codon to a Leu codon at aa279, a Ser to Phe at aa249, a His to Tyr at aa196, and a Pro to a Leu at aa156 in the NdhB protein. NdhB is one of the eleven chloroplast-encoded subunits of the chloroplast NDH complex. We hypothesized *ndhB* editing defects observed in *ocp3* plants would affect encoded protein function, which in turn would alter photosynthetic parameters in the mutant. NDH complex activity can be monitored as a transient increase in chlorophyll fluorescence reflecting plastoquinone pool reduction after turning off actinic light, as originally demonstrated by Shikanai et al. [11]. Figure 5A shows a typical chlorophyll fluorescence trace from Arabidopsis Col-0 and its comparison with *crr21*, a mutant lacking NDH activity. In *ocp3*, the post-illumination increase in chlorophyll fluorescence was modified in a manner similar to what occurred in *crr21* plants, indicating that NDH activity was compromised. This result strongly indicated OCP3 is a chloroplast factor pivotal in normal NDH complex function. This important phenotype was confirmed by employing additional mutant alleles. Due to the absence of T-DNA insertions mutants for the *OCP3* locus, and being the *ocp3* mutant currently used a loss-of-function EMS mutant, we generated additional mutant alleles of this gene by artificial microRNA (amiRNA) interference. Two independent homozygous amiRNA lines (i.e. amiRNA-2 and amiRNA-3) phenocopying the original *ocp3* mutant (Figure S5A–D), were selected. These lines were designated *ocp3-2* and *ocp3-3*, respectively, and the original *ocp3* now designated *ocp3-1*. Defective NDH activity was recorded in these mutants (Figure 5A). Complementation of *ocp3-1* plants with an OCP3 wild-type sequence fully restored the post-illumination increase of chlorophyll fluorescence (Figure 5A). These results confirmed the importance of OCP3 for appropriate NDH complex function.

RNA editing results in amino acid changes that directly alter protein translation, function, or even may act to destabilize multiprotein complexes. The NDH complex is unstable when NdhD subunit is absent due to editing-mediated translation defects [34], [35]. The NdhB subunit defects observed in *ocp3* plants were evaluated to determine the effects on NDH stability *in vivo*. Protein blots were analyzed using antibodies against the NdhI and NdhJ subunits, which served to monitor NDH complex stability (Figure 5B). NdhI and NdhJ accumulation levels did not experience noticeable changes in *ocp3* mutants compared to Col-0 plants. Similarly, NDH complex stability remained intact in *crr21* plants (Figure 5B). Although the exact function and organization of the whole set of subunits of the NDH complex in plants remains to be fully elucidated [36], our results indicate that the four amino acid residues in the NdhB subunit which were derived from editing-mediated codon conversion appear important for activity, but not for assembly of the NDH complex.

### Chloroplast NDH activity-defective mutants show enhanced disease resistance

**Figure 6:**
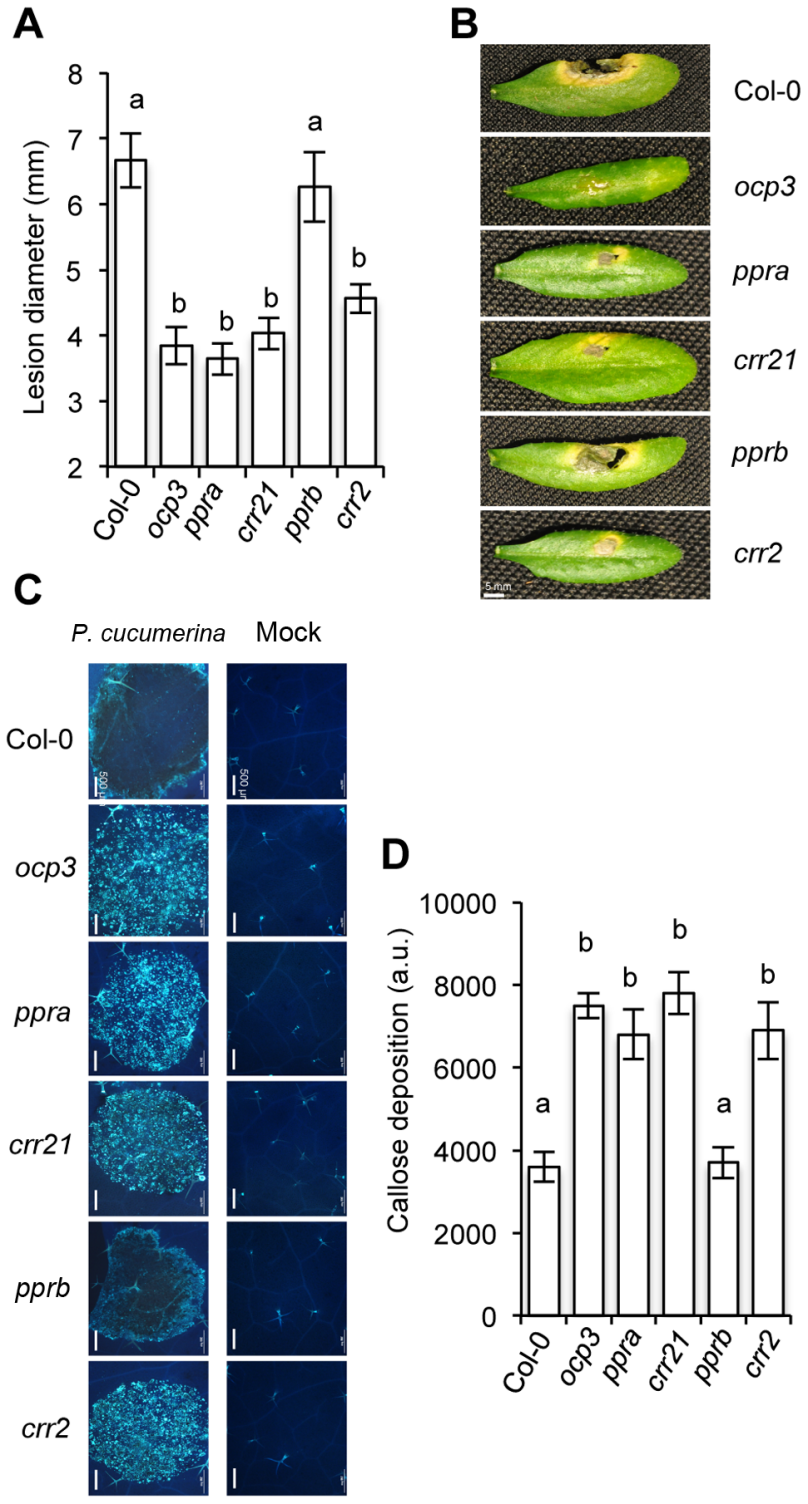
Comparative immune responses of plastid PPR-related mutants to inoculation with *P. cucumerina*. (**A**) *crr2*, *crr21*, *ppra*, and *pprb* disease resistance responses to *P. cucumerina* compared with *ocp3* and wild-type (Col-0) plants. Lesion diameter of 20 plants per genotype and four leaves per plant were determined 12 d following inoculation with *P. cucumerina*. Values are means and ± SE (n = 80). ANOVA detected significant differences at the P<0.05 level. Experiments were repeated three times with similar results. (**B**) Representative leaves from each genotype at 12 days following inoculation with *P. cucumerina*. Bar represents 5 mm. (**C**) Aniline blue staining and epifluorescence microscopy was applied to visualize callose accumulation. Micrographs indicate *P. cucumerina* inoculation and infection site in the different *Arabidopsis* genotypes at 0 h.p.i (right panel) and at 48 h.p.i. (left panel). Bar represents 500 µm. (**D**) The number of yellows pixels (corresponding to pathogen-induced callose) per million on digital photographs of infected leaves were used as a means to express arbitrary units (i.e. to quantify the image) at 48 h.p.i. Data are visible microscopy averages from Col-0 and mutant plants (±SE). Different letters above bars indicate statistically significant differences between genotypes, according to one-way ANOVA (P<0.05, n = 15).

We hypothesized that via NDH complex inhibition, plants could develop an alerted immune status. This might explain why *ocp3* plants exhibited enhanced disease resistance to fungal pathogens resulting from earlier and more intense callose synthesis and deposition following pathogen exposure [25], [26]. If so, then mutants showing similar chloroplast NDH complex defects would activate the same immune status, and become resistant to fungal attack. Consequently, we challenged *crr21* and *crr2* mutants with *P. cucumerina*, and studied disease susceptibility in comparison to the resistant *ocp3* plants, and the susceptible Col-0 plants. CRR2 is a distinct PPR protein that functions in the intergenic RNA cleavage between *rps7* and *ndhB*, which is essential for subunit B translation, and *crr2* mutants are compromised in NDH activity [35]. *ppra*, a previously uncharacterized T-DNA mutant, defective in the expression of *PPRa* (Figure S8) encoding a PPR protein of unknown function that is highly co-expressed with *CRR21* and *OCP3* (Figure 3A), was also evaluated. Similarly, *pprb*, a T-DNA mutant defective in another co-expressed PPR of unknown function (Figure S7) was included in these experiments for comparison. Following inoculation with *P. cucumerina*, disease was scored 12 d after inoculation by following necrosis and chlorosis extent present in inoculated leaves. As expected, Col-0 plants were highly susceptible to *P. cucumerina*, and all inoculated plants showed extended necrosis accompanied by extensive proliferation of fungal mycelia (Figure 6A–B). The same disease susceptibility was observed in the *pprb* mutant, indicating this *PPR* gene is not essential in plant's defense activation (Figure 6A–B). In marked contrast, the inoculated *crr21*, *crr2*, and *ppra* plants responded with a substantial increase in disease resistance to *P. cucumerina* infection that was of a magnitude similar to that attained in *ocp3* plants (Figure 6A–B). Comparative cytological observations were performed at the sites of attempted fungal infection and the degree of induced callose deposition induction in inoculated leaves was monitored after staining with aniline blue, and examination by fluorescence microscopy. Results indicated none of the mutants exhibited aniline blue staining in control leaves (Figure 6C). Col-0 and *pprb* plants deposited callose locally at sites demarcating the zones of extended fungal growth. In marked contrast, *crr21*, *crr2*, *ppra*, and *ocp3* plants all exhibited intensified and highly localized callose deposition in response to fungal infection, which occurred at zones where fungal growth and colonization was impeded (Figure 6C–D). Consequently, heightened disease resistance, and increased callose deposition were concurring traits in mutants defective in the correct editing of RNAs encoding subunits of the chloroplast NDH complex.

### Fungal infection interferes with editing in plastids

The above results indicated that editing efficiency, chloroplast NDH activity, and disease resistance to fungal pathogens are linked traits mediated by OCP3. Fungal infection provokes local down regulation of *OCP3* in wild type plants [25], therefore we hypothesized that following Col-0 inoculation with a fungal pathogen, editing inhibition of *ndhB* would very likely arise and, the NDH complex would consequently be affected. Therefore, we inoculated Col-0 plants with the fungal pathogen *P. cucumerina* and examined the editing status of RNAs corresponding to chloroplast-encoded subunits of the NDH complex (i.e, NdhB, NdhF, NdhG and NdhD) by bulk sequencing of RT-PCR products. We identified major alterations in the RNA edition of *ndhB*. Eight sites normally edited in the *ndhB* transcripts (i.e. ndhB-1 to ndhB-8) showed inhibition at 48 h.p.i. with *P. cucumerina* (Figure S6). Results for the other NDH subunit RNAs indicated only the editing status of *ndhD* transcript was notably affected, and only at position 117166 (ndhD-1 site) which controls NdhD translation (Supplemental Figure S6). These results surpass the four distinct defective editing sites identified in the *ocp3* mutant (Figure S6 and Figure 4). Therefore, in addition to OCP3, other factors appeared to be targeted for the realization of the fungal-promoted editing inhibition.

**Figure 7:**
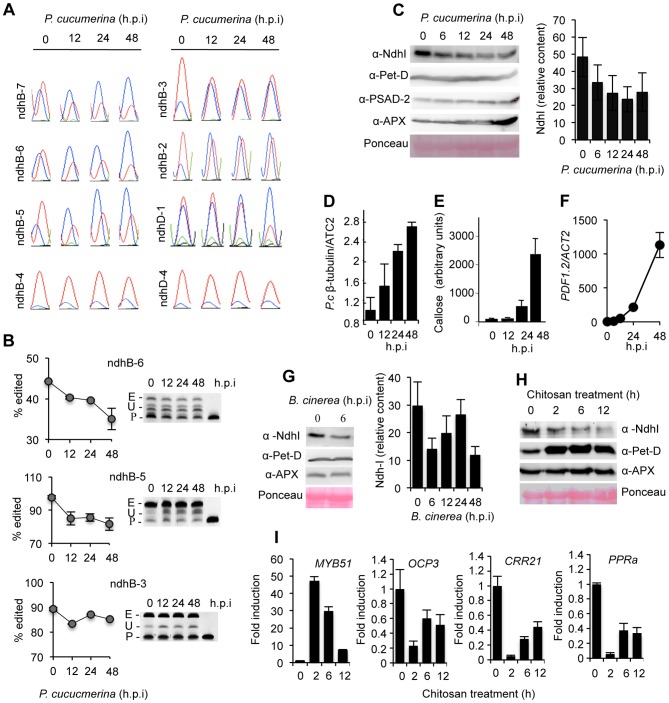
Pathogen-triggered editing inhibition in plastid *ndhB* and *ndhD* transcripts. (**A**) Portion of the electrophoretograms from RT-PCR bulk sequencing corresponding to the editable cytosine residue at sites ndhB-7, ndhB-6, ndhB-5, ndhB-4, ndhB-3, ndhB-2, ndhD-1, and ndhD-4 are shown for Col-0 plants at 0,12, 24 and 48 h post-inoculation with *P. cucumerina*. (**B**) PPE assays following fungal infection for ndhB-6, ndhB-5 and ndhB-3 sites confirms the reduction of editing extent as detected by bulk sequencing. The PPE products run on acrylamide gels are shown on the right. E, edited; U, unedited; P, primer. (**C**) Immunoblot analysis of NDH subunit I (NdhI), subunit IV of cytochrome *b6f* (Pet-D), PSI subunit D-2 (PSAD-2) and ascorbate peroxidase (APX) from Co-0 plants at 0, 6, 12, 24 and 48 h following inoculation with *P. cucumerina*. Intensity of NdhI immunoreactive bands was quantified and plotted on the right graph. Bars represent mean ± SD, n = 3 independent replicates. The experiment was repeated three times with similar results (**D**) Extent of *P. cucumerina* growth on inoculated leaves. At the times indicated DNA was extracted from leaves and the amount of the *P. cucumerina β-tubulin* gene quantified by qPCR. Data are standardized for the presence of the *P. cucumerina β-tubulin* gene in Col-0 at time 0. Data represent the mean ± SD; n = 3 biological replicates. (**E**) Determination of *P. cucumerina*-induced callose deposition in inoculated leaves of Col-0 plants. (**F**) *P. cucumerina*-induced expression of the defense-related *PDF1.2* gene as determined by RT-qPCR. Data represent the mean ± SD; n = 3 biological replicates. (**G**) Reduction of NdhI subunit content following inoculation of Col-0 with *B. cinerea*. NdhI content was quantified as in (C). On the right a Western blot detail revealing early (at 6 h.p.i) fungal-induced down regulation of NdhI subunit accumulation is shown. (**H**) Early induced down-regulation of NdhI protein accumulation in Arabidopsis seedlings by chitosan. Chitosan was applied for the times indicated to Arabidopsis seedlings and proteins analyzed by Western blot with anti-NhHI, anti-Pet-D and anti-APX antibodies. The experiment was repeated three times with similar results. (**I**) Chitosan-induced gene activation of *MYB51* and concomitant gene repression of *OCP3*, *CRR21* and *PPRa* as determined by RT-qPCR. Data represent the mean ± SD; n = 3 biological replicates.

Temporal recording in a time course experiment following *P. cucumerina* inoculation revealed that editing inhibition is an early plant response to fungal attack. Most inhibition changes at the identified pathogen-sensitive *ndhB* editing sites were induced early following *P. cucumerina* inoculation (at 12 h.p.i.), and were sustained up to 48 h.p.i (Figure 7A), indicating the special vulnerability of *ndhB* editing to pathogenic cues. Results showed the specific editing defects at the ndhD-1 site lagged behind *ndhB* editing inhibition, reaching maximal inhibition at 48 h.p.i (Figure 7A). Some of these early effects were further corroborated by specific PPE assays, which provided estimates that editing at ndhB-6, ndhB-5, and ndhB-3 sites were inhibited following pathogen inoculation at different efficiencies and declining rates. ndhB-6 editing inhibition was the most prominent, with an efficiency that abruptly dropped at 12 h.p.i. and progressively decayed thereafter (Figure 7B).

### Early chloroplast NDH complex destabilization is part of the immune response

Following these previous observations we asked if stability of the NDH complex could become also altered following fungal infection. To assess this, NDH complex stability was monitored by Western blots using antibodies against one of the NDH subunits (i.e. NdhI). We observed NdhI accumulation level decayed very early following pathogen inoculation, with apparent reduction occurring at 6 h.p.i. (Figure 7C). The decay was progressive and showed an approximate 50% reduction in NdhI protein at 24 h.p.i. (Figure 7C). The results suggested decay specificity for NDH complex protein, since chloroplast integrity, measured using other marker proteins (i.e., Pet-D, PSAD-2 and APX), did not change or even increase in response to the fungus (Figure 7C). The decay process was set in motion at early stages of infection, and was inversely correlated with fungal growth (Figure 7D). Furthermore, the observed pathogen-triggered dismantling of the NDH complex subunit preceded the activation of other plant responses, which are diagnostic of an activated plant immune response. Deposition of the cell wall β-1,3-glucan polymer callose, identified and quantified following aniline blue staining of inoculated leaves, clearly lagged behind observed editing defects and dismantling of NDH complex (Figure 7E). Similarly, transcriptional activation of the defense related gene *PDF1.2* followed editing defect accumulation (Figure 7F). Furthermore, the inhibitory effect on NdhI accumulation was mirrored by *Botrytis cinerea* inoculation, another fungal pathogen (Figure 7G). The decay in NDH subunit content promoted by *B. cinerea* (Figure 7G, right graph) was notable, but not as progressive as observed in *P. cucumerina*, presumably reflecting different infection styles for the two distinct fungal pathogens.

The rapid editing inhibition and the parallel dismantling of the NDH complex constitutes two early chloroplast responses to pathogens, evoking integration of these processes as part of the mechanism governing immune response activation. Therefore, we verified if editing inhibition and NDH complex destabilization could be similarly triggered by application of chitosan (2-amino-2-deoxy-(1-4)-β-D glucopiranan), a naturally-occurring pathogen-associated molecular pattern (PAMP) compound able to elicit plant innate immune responses similar to those activated by complex fungal pathogens [37]. Results showed strong and rapid (within 2 h) down-regulation of NDH subunit accumulation was promoted by the sole application of chitosan to wild type Arabidopsis seedlings (Figure 7H). Interestingly, we observed also an early and abrupt down-regulation of *OCP3*, *CRR21* and *PPRa* gene expression following chitosan treatment, which contrasted with the concurring high transcriptional activation of *MYB51* (Figure 7I), the latter a transcription factor required for PAMP-triggered callose deposition in Arabidopsis [38]. Since CRR21, OCP3 and PPRa are nuclear-encoded chloroplast factors required for an effective plant immune response (Figure 6), the results indicate the existence of a nuclear PAMP-mediated transcriptional regulation of NDH complex-related editing regulatory genes as an integral component of innate immunity. This thus represents an additional layer of control of chloroplast NDH complex activity interconnecting nuclei and chloroplasts. Moreover, electrophoregrams comparison of bulk sequencing of RT-PCR products, generated at different times after chitosan treatment, revealed that chitosan-induced NDH complex dismantling was also accompanied by an early editing inhibition at seven *ndhB* editing sites, and at three *ndhD* editing sites (Figure S7). Therefore, these results provided additional support for the engagement of plastid RNA editing inhibition in plant immunity. Moreover, the observed rapid and transient dismantling of the NDH complex that follows perception of pathogenic cues suggests the engagement of a highly regulated proteolytic system in the chloroplast. The identification and characterization of such proteolytic system remains a challenge for the future.

## Discussion

This study provides new insights into the control of disease resistance in plants, and reinforces the importance of the chloroplasts in plant immunity. Our data identified OCP3 targeted to chloroplast, a finding that conceptually changed the previous assumption that OCP3 could function as a nuclear transcription factor. Furthermore, confocal microscopy revealed that OCP3 accumulated in plastids matching several PPR proteins. Moreover, *OCP3* was found to be closely co-expressed with a cluster of 9 genes encoding PPR proteins including *CRR21*. CRR21 is responsible for site 2 editing at *ndhD* transcript, and *ndhD* encodes the D subunit of the chloroplast NDH complex [13], a crucial component of the CEF machinery around PSI [11]. In *crr21* plants, NDH complex activity is impaired and CEF activity compromised [13], supporting a predominant post-transcriptional level of control. All these observations prompted us to hypothesize that OCP3 was involved in RNA editing in plastids. Therefore, we performed a comparative systematic study of the editing status of chloroplast transcripts between Col-0 and different *ocp3* mutants. This study revealed that OCP3-defective plants carry specific editing defects at ndhB-6, ndhB-4, ndhB-3, and ndhB-2 sites. The observation that OCP3 associates *in vivo* with the *ndhB* transcript, as revealed by RIP assays, reinforce the consideration that OCP3 contributes to control over the extent of *ndhB* transcript editing. However, OCP3 appears not to carry any structural motif resembling the conserved RNA recognition motif (RRM), not even the motifs characteristic of other proteins functioning as *trans*-factors essential for editing, such as those present in the large subclasses of the pentatricopeptide repeat (PPR)-containing family proteins [13], [14]. This may suggest that the association of OCP3 with the *ndhB* RNA molecule may be likely indirect, presumably though the interaction with canonical RNA binding proteins recognizing appropriate *cis*-elements present in the *ndhB* RNA molecule such as those RNA-binding proteins mentioned above. Therefore, OCP3 may serve a regulatory role on the editing apparatus by regulating and/or adjusting the editing extent of the *ndhB* transcript according to external environmental cues. This appears to be the case also for other described editing accessory proteins such as the recently identified multiple organellar RNA editing factor (MORF) and members of the RNA-editing interacting protein (RIP) family [39], [40].

*ndhB* encodes the NDH complex B subunit, therefore we next hypothesized that the absence of a functional OCP3 protein should result in a defective NDH complex. Results indicated that the observed alterations in *ndhB* editing in *ocp3* plants affected NDH activity but not NDH complex stability. This is a feature also found in other editing-related mutants (i.e. *crr21*). However, in other cases, the defective gene results in lack of NDH complex accumulation, as seen when editing defects impedes translation initiation of NdhD subunit (i.e. *crr4*, [10]) or when appropriate maturation of *ndhB* transcript is blocked (i.e. *crr2*, [35]). The editing defects in *ocp3* plants resulted in an inactive NDH complex that compromised normal CEF around PSI. We therefore concluded that OCP3 is an integral plastidial factor required for fine-tuning CEF around PSI, and this control is exerted post-transcriptionally through the regulation of *ndhB* transcript editing. This finding has important consequences as it represents the first evidence interfacing plant immunity, RNA editing and CEF. Therefore, one can propose that when OCP3 fails, as occurs in *ocp3* plants, then accurate *ndhB* transcript editing is impeded, and in turn NDH complex is altered and eventually CEF inhibited. Since in chloroplast the NDH complex is considered to alleviate various oxidative stresses [41], [42], it can be speculated that a defective CEF pathway could generate ROS locally that eventually may resulted in disease resistance activation. Since *ocp3* plants carry constitutive enhanced production of ROS species, particularly H_2_O_2_, and also show constitutive expression of ROS-inducible genes [25], [28], and *OCP3* gene expression was rapidly down regulated following fungal attack [25], we reasoned that editing will fail, and consequently NDH impaired, when a plant encounters a pathogen. This led us to find that Arabidopsis respond to attempted *P.cucumerina* infections by activating a rapid mechanism of editing inhibition which affected the 8 major editing sites in *ndhB*, therefore including those requiring OCP3, plus an additional editing site at *ndhD*. This suggests involvement of other factors functioning as pathogen-sensitive regulators of the editing process. In fact, a similar cause-effect relationship was observed in *crr2*, *crr21 and ppra* plants, which exhibited the same response as *ocp3* plants when inoculated with *P. cucumerina*. Their characteristic heightened disease resistance was accompanied by increased callose deposition in response to fungal infection, evoking activation of a mechanism for priming of callose deposition in these mutants similar to that previously discovered in *ocp3* plants [26].

In wild type plants, in addition to the pathogen-induced editing inhibition of *ndhB* transcripts, we observed the NDH complex becoming rapidly destabilized and therefore dismantled, presumably by the action of chloroplast proteases. Therefore, either NDH complex activity and/or stability constitute distinct hallmarks of the plant's defense response to fungal pathogens. Whether or not editing inhibition and NDH complex stability are linked processes or rather represent independent processes remains unknown and is a challenging issue for future research. Furthermore, strong repression of *OCP3* and *CRR21* gene expression, severe editing inhibition of *ndhB* and *ndhD* transcripts, and NDH destabilization were also rapidly triggered by the sole application of chitosan, which functions as a PAMP mimicking fungal structures. Consequently, editing inhibition and dismantling of the NDH complex appeared definitively engaged during activation of innate immunity. Therefore, when appropriately and timely activated following pathogen perception, the mechanisms leading to alteration of the NDH complex in the chloroplast should serve to set in motion a signaling process leading to an effective defense response to halt the advance of the pathogen.

Cumulatively, these observations reinforced the idea that maintaining NDH complex integrity is pivotal to normal CEF functioning during photosynthesis, however its timely inhibition following pathogen attack is fundamental for plant immunity. Therefore, modulation of the NDH complex activity must be under a delicate balance, requiring precise but flexible control. The breadth of our data indicate control occurs at the RNA editing level, a process where the described proteins ultimately serve as sensors regulating the rate of NDH complex activity. Therefore, OCP3, and presumably those PPRs and accessory proteins mediating editing extent of NDH complex subunits, exhibit a Janus-faced function, serving reciprocally as negative regulator of plant immunity and as positive regulator of CEF during oxygenic photosynthesis.

## Materials and Methods

### Plants growth conditions

*Arabidopsis thaliana* plants were grown in a growth chamber (19–23°C, 85% relative humidity, 100 mEm^−2^ sec^−1^ fluorescent illumination) on a 10-hr-light and 14-hr-dark cycle. All mutants are in Col-0 background.

### Gene constructs and transgenic lines

For the *OCP3-GFP, -YFP and -mCHERRY* constructs, the *OCP3* full length cDNA was amplified by PCR using *Pfu* DNA polymerase (Stratagene, San Diego, CA) and specific primers including Gateway adapters, and recombined into pDONR221/207 using BP ClonaseMixII kit (Invitrogen). After sequencing, all constructs were recombined into pEarleyGate101 destination vector using LR ClonaseMixII kit (Invitrogen) and introduced into *ocp3* plants for complementation analysis or when indicated in Col-0 via *Agrobacterium* transformation. Cloning of the different ORFs employed in the present work and their fusion with the indicated fluorescent tag was done is a similar way. List of primers used for cloning purposes is provided in Supplementary information.

### Chlorophyll fluorescence analysis

Chlorophyll fluorescence was measured using a MINI-pulse-amplitude modulation portable chlorophyll fluorometer (Dual-PAM-100, Walz, Effeltrich, Germany). The transient increase in chlorophyll fluorescence after turning off actinic light (AL) was monitored as described [12].

### Confocal laser-scanning microscopy

Plant tissue was observed with a Leica TCS LS spectral confocal microscope using and HCX PL APO ×40/1.25-0.75 oil CS objective. GFP- or YFP-derived fluorescence was monitored by excitation with 488- and 514-nm argon laser lines, respectively, and emission was visualized with a 30-nm-width band-pass window centered at 515 nm. When RFP and CHERRY were used, excitation was performed by means of a 543-nm green-neon laser line, and fluorescence emission was collected at 695 to 630 nm.

### OCP3 amiRNAs

The artificial microRNA designer web WMD3 (http://wmd3.weigelworld.org/cgi-bin/webapp.cgi) was employed for designing the 21 mer amiRNA sequence specific for OCP3 (At5g11270) and for subsequent cloning and amplifications protocols. The target region selected in *OCP3* was 5-′GCGTCGTAAAACTAGTATTAA-3 (positions 625 to 645) and the 4 oligonucleotide sequences used to engineer the artificial miRNA into the endogenous miR319a precursor by site-directed mutagenesis were:

- miR-s TTAATACTAGTTTTACGGCGCtctctcttttgtattccaa,
- miR-a GCGCCGTAAAACTAGTATTAAtcaaagagaatcaatgatc,
- miR*s GCACCGTAAAACTTGTATTATtcacaggtcgtgatatgat,
- miR*a ATAATACAAGTTTTACGGTGCtctacatatatattcctaa,

As a template for the PCRs the pRS300 was used. The amiRNA sequence was cloned behind a 35S gene promoter and the binary vector used to transform Arabidopsis Col-0 plants. Eight independent transformants were initially selected and two homozygous lines showing remarkable reduced expression (amiRNA-2 and amiRNA-3) of *OCP3* were selected for further studies.

### RNA extraction, RT, qPCR, HRM and PPE

Total RNA was extracted using TRIzol reagent (Invitrogen) following the manufacturer's recommendations and further purified by lithium chloride precipitation. For reverse transcription, the RevertAid H Minus First Strand cDNA Synthesis Kit (Fermentas Life Sciences) was used. Quantitative PCR (qPCR) amplifications and measurements were performed using an ABI PRISM 7000 sequence detection system, and SYBR-Green (Perkin-Elmer Applied Biosystems). *ACTIN2/8* was chosen as the reference gene. All 34 known Arabidopsis chloroplast RNA editing C targets were assayed by high resolution melt (HRM) as described [7]. Chloroplast RNA editing sites were assayed by RT-PCR bulk sequencing using similar set of primers. Primers for amplicons covering each of the 34 editing sites present in plastids are listed below in Supplemental information. Poison primer extension (PPE) analysis on chloroplast sites were conducted as described [43] and sequence of the corresponding fluorescent primers used are listed below in Supplemental information.

### Transient expression in protoplasts

Protoplasts isolation and transfection protocol with the different gene constructs was as described [44].

### Western blots

Protein crude extracts were prepared by homogenizing ground frozen leaf material with Tris-buffered saline (TBS) supplemented with 5 mM DTT, protease inhibitor cocktail (Sigma-Aldrich). Protein concentration was measured using Bradford reagent; unless otherwise indicated 20 µg of total protein was separated by SDS-PAGE (12% acrylamide w/v) and transferred to nitrocellulose filters. The filter was stained with Ponceau-S after transfer, and used as a loading control. Unless otherwise indicated, immunoblots were incubated with the indicated primary antibodies at the appropriate dilution and developed by chemiluminescence using an anti-IgG peroxidase antibody (Roche) at a 1∶1000 dilution and Western Lighting plus-ECL substrate (Perkin-Elmer).

### N-terminal sequencing

Protein samples resolved by SDS-PAGE were blotted to PVDF membranes and the band of interest identified by Ponceau staining. The band sector corresponding to OCP3 was recovered and subjected to five/six cycles of automated microsequencing by sequential Edman degradation in an Applied Biosystems, Procise 494.

### Botrytis cinerea and *Plectosphaerella cucumerina* bioassays

In both *B. cinerea* and *P. cucumerina* infections, five-week-old plants were inoculated as described [25], [26], with a suspension of fungal spores of 2.5×10^4^ and 5×10^6^ spores/mL respectively. The challenged plants were maintained at 100% relative humidity. Disease symptoms were evaluated by determining the lesion diameter of at least 50 lesions 3 or 12 days after inoculation. For pathogen-induced callose deposition analyses, infected leaves were stained with aniline blue and callose deposition quantifications were performed as described by Garcia-Andrade *et al*. [26].

### Chitosan treatments

Approximately 15 sterilized Col-0 seeds were sown per well in sterile 12-wells plates, containing filter-sterilized MS mediums without Gamborg's vitamins and with 0,5% of sucrose. Seedlings were cultivated under standard growth conditions (15 h day cycle; 20°C/17°C) with a light intensity of 150 µM/m2/s. After 7 days, the growth medium was replaced by fresh MS medium. One day later, plants were mocked or challenged with chitosan at the final concentration of 10 µg/mL in the growth medium, and at the indicated times the samples were collected and immediately frozen in liquid nitrogen.

### Chloroplast fractionation

Fractionation of total chloroplasts preparations from full expanded Arabidopsis leaves into stromal, thylakoids and membrane envelope was performed as described [45]. Each suborganellar fraction was identified and validated by developing Western blots with anti-BCCP (stroma), anti-NIP (thylakoids) and anti-OEP21 (membrane envelope) antibodies. Antibodies were obtained from Uniplastomic (Gieres, France).

### RNA Immunoprecipitation (RIP) followed by RT-PCR

RIP assays were performed as described [46] with minor modifications. Essentially, 2 g of leaf tissue from Arabidopsis plants (4 weeks old plants) were ground to a fine powder with a mortar and pestle in liquid nitrogen and homogenized in 12.5 mL/g lysis buffer (50 mM Tris-HCl, pH 7.4, 2.5 mM MgCl_2_, 100 mM KCl, 0.1% Nonidet P-40, 1 µg/mL leupeptin, 1 µg/mL aprotonin, 0.5 mM phenylmethylsulfonyl fluoride, one tablet of Complete proteinase inhibitor tablet (Roche), and 50 units/mL RNase OUT (Invitrogen). Cell debris was pelleted by centrifugation for 5 min at 12,000 rcf at 4°C. Clarified lysates were incubated with 4 µg/mL of anti-HA antibody (Roche) for 15 min at 4°C and then with 100 µL of Protein-A agarose (Roche) per milliliter for 30 min at 4°C. Beads were washed six times for 10 min with lysis buffer at 4°C and then divided for protein and RNA analysis. RNAs were recovered by incubating the beads in 0.5 volumes of proteinase K buffer (0.1 M Tris-HCl, pH 7.4, 10 mM EDTA, 300 mM NaCl, 2% SDS, and 1 µg/µL proteinase K (Roche)) for 15 min at 65°C, extraction with saturated phenol, phenol∶chloroform∶isoamyl alcohol and chloroform, and ethanol precipitation. For RT-PCR assays, 1 µg of total RNA was used for the input fraction, and 20% of the RNA immunoprecipitate was used for the immunoprecipitation. PCR to amplify fragments corresponding to *ndhB* and *ndhD* cDNAs was done using specific oligos, CHLORO 187 FW/RV and CHLORO 212 FW/RV, respectively, as listed in Supplemental material. For protein blot assays, 10 µL of clarified eluate was loaded for the input fraction, and 3% of the immunoprecipitated beads was used for the immunoprecipitation. OCP3:GFP:HA was detected by immunoblotting and chemiluminescence using and anti-GFP peroxidase antibody (Roche) at a 1∶1000 dilution and Western Lighting plus-ECL substrate (Perkin-Elmer).

### T-DNA Arabidopsis mutants

Homozygous lines of *ppra* and *pprb* T-DNA insertion mutants were identified by PCR using primers listed in Supplemental information.

## Supporting Information

Figure S1 **Protein localization in** ***N. benthamiana*** **protoplast.** Fluorescent confocal microscopy evaluation of protein localization in transfected protoplast of *N. benthamiana* with a 35S::YFP construct (upper panel), a *35S::YFP-OCP3* construct (middle panel) and a *35S::OCP3-YPF* constructs (lower panel). YFP-specific fluorescence is shown in green and chlorophyll-derived fluorescence is shown in red.
(TIF)

Figure S2 **Functional characterization of the signal peptide sequence of OCP3.** (A) SDS-PAGE and protein immunoblot with anti-GFP antibody indicating protein band position of OCP3-GFP precursors (Polyp1 and Polypep2) that were used for N-terminal amino acid sequence determination by Edman sequential degradation. Five rounds of degradation were conducted for each polypeptide which rendered the indicated 5 amino acid long N-terminal sequence. (B) Scheme depicting the different gene construct used for testing functionality of the OCP3 signal peptide. Green: YFP protein; white: OCP3 protein; red symbol: relative position where the Δ_68–74_ internal deletion in the signal peptide sequence was created. (C) Confocal microscopy localization of the relevant constructs shown in (B). (D) Comparative complementation test of the *ocp3* mutant (which show constitutive GUS expression as driven by the Ep5C gene promoter) with construct *35S::OCP3-YFP* and construct *35S::OCP3_68–74_-YFP*. The latter carries a deletion of 7 amino acids in the Signal peptide of OCP3 and was unable to be processed in the chloroplast. (E) Western blot with anti-GFP antibodies of proteins extracts derived from *ocp3* plants transformed with *35S::OCP3-YFP* and *35S::OCP3_68–74_-YFP* gene constructs. (F) Western blots of the indicated chloroplast compartments obtained from chloroplasts preparations derived from *ocp3* plants transformed with the *35S::OCP3-YFP* gene construct. Westerns were developed using anti-GFP; anti-BCCP1 (as marker for the stroma (lane 1; loaded with 4 µg total protein)); anti-OEP21 (as marker for membrane envelop (lane 2; loaded 1 µg total protein)); and anti-NIP (as marker for thylakoids (lane 3; loaded with 1 µg total protein).
(TIF)

Figure S3 **Co-expression gene vicinity network for OCP3.** Nodes indicate individual genes, and edges indicate whether two genes are co-expressed above a certain mutual rank. Red, yellow, green, and grey nodes indicate whether mutations in the gene cause embryo lethality (red), gametophytic lethality (yellow), any other biological phenotype (green), or if no mutant phenotype currently is available (grey) according to TAIR. The color edges indicate strength of the coexpression based on mutual rank relationships between the individual gene pairs. Green, orange, and red edges indicate a mutual rank relationship ≤10 (green), between 11 and 20 (orange) and 21 and 30 (red), respectively, for each connected gene. The network was generated, and modified from AraGenNet (http://aranet.mpimp-golm.mpg.de/aranet; Mutwil et al., 2010).
(TIF)

Figure S4 **Analysis of the editotype of the** ***ocp3*** **mutant as revealed by high resolution melting (HRM).** (**A**) Editing of the current 34 sites in *A. thaliana* chloroplast transcripts. Editing regulated genes are shown in the first column. The exact positions in the chloroplast genome sequence of each edited nucleotide is shown in the next column. Observed changes by HRM between Col-0 and *ocp3* plants are marked with (+) symbols, and absence of differences are marked with (−) symbol. The four editing sites found to be affected in *ocp3* plants are dashed in grey. (**B**) Example of HRM analysis, monitored by decrease in fluorescence as the temperature increase, for the amplicon encompassing *ndhB* transcript at position 95608 (ndhB-7 site) which suffers no variation between Col-0 and *ocp3*. (**C**) Examples of HRM analysis where the presence of less thermostable heteroduplexes in a sample alters the shape of the melting curves such as occurs with amplicons for transcript *ndhB* at positions 95644 (dnhB-6 site), 96579 (ndhB-3 site) or 96698 (ndhB-2 site) which suffer variation between Col-0 and *ocp3*.
(TIF)

Figure S5 **Characterization of Arabidospis strains silenced in** ***OCP3*** **and generated artifitial microRNAs (amiRNAs).** The new *ocp3* mutant strains (*ocp3-2/amiRNA-2* and *ocp3-2/amiRNA-3*) were generated by artifitial microRNAs (amiRNAs) designed to specifically target and down-regulate *OCP3*. The resulting mutant phenotypes were compared to the EMS-induced *ocp3-1* mutant and Col-0. (**A**) Comparison of vegetative growth and anatomical appearance between Col-0, *ocp3-1*, *ocp3-2* and *ocp3-3*. (**B**) RT-qPCR of *OCP3* transcript levels in the four indicated genetic backgrounds. *OCP3* expression was normalized to *ACTIN2*.8 expression. Bars represent mean ± SD, n = 3 independent replicates. (**C**) Lesion size resulting from *P. cucumerina* infection in Col-0, *ocp3-1*, *ocp3-2*, and *ocp3-3* plants at 12 days post-inoculation. Values are means and ± SE (n = 50). Asterisks indicate significant differences (LSD test; P<0.05). (**D**) Sequence electrophoregrams corresponding to the RNA editing sites of ndhB-6 (95644), ndhB-5 (95650), ndhB-4 (96419), ndhB-3 (96579), ndhB-2 (96698) as derived from bulk RT-PCR sequencing of amplicons from Col-0, *ocp3-1*, *ocp3-2*, and *ocp3-3* plants mRNA preparations. Editing sites are indicated by a red T residue and unedited sites by a red C residue. Partial editing inhibition is indicated by red T/C.
(TIF)

Figure S6 ***P. cucumerina*** **-mediated editing inhibition.** Nucleotide sequences of RT-PCR products obtained from Col-0 plants at 0 hours and at 48 hours post-inoculation with *P. cucumerina*, and its comparison with non-inoculated *ocp3-1* plants, are shown as sequencing electrophoregrams. Editing sites for the four transcript encoding the chloroplast-encoded NDH complex subunits (i.e., NdhB, NdhD, NdhF, and NdhG) are indicated by arrows pointing to the corresponding peaks. Observed editing inhibition following *P. cucumerina* infection are marked with a blue cross above the corresponding editing site.
(TIF)

Figure S7 **Sequencing electrophoregrams of nucleotide sequence of RT-PCR products obtained from Col-0 seedlings at the times indicated following mock or chitosan (10 µg/mL) treatment.** The electrophoregrams show the C nucleotide either edited or not edited at the corresponding editing site of the corresponding transcript. Shown are editing sites for which chitosan exerts editing inhibition effect.
(TIF)

Figure S8 ***PPRa*** **(At4g2119) and** ***PPRb*** **(At4g3082) T-DNA insertion mutants.** (**A**) The *ppra* mutant (strain Salk-007827) carries a T-DNA insertion at 340 nt upstream of the ATG initiation codon and therefore could affect expression of the gene. (**B**) The *pprb* mutant (strain Salk-204171) carries a T-DNA insertion internal to the unique exon, close to the ATG initiation codon, and therefore disrupts the ORF. Exons are indicated with solid rectangles. T-DNA insertions are indicated with white rectangles. (**C**) None of the mutations affect the normal growth of the plants and both mutants resemble Col-0 plants in morphological phenotype. (**D**) RT-qPCR of *PPRa* transcript levels in Col-0 and in *ppra* mutant reveal that expression of *PPRa* was down-regulated in the mutant. *PPRa* expression was normalized to *ACTIN2*.8 expression. Bars represent mean ± SD, n = 3 independent replicates.
(TIF)

Table S1 **Genes ID co-expressed with** ***OCP3*** **in cluster 59, their functional annotation and MapMan classification term derived from cluster 59, as appearing in the gene network generated using the AraGenNet platform (** http://aranet.mpimp-golm.mpg.de/aranet **; Mutwil et al., 2010).**
(XLSX)

Table S2 **Genes showing highest co-expression index in relation to** ***OCP3*** **(see Table S1), with their corresponding ID, functional annotation, and MapMan term. **The PPRs encoding genes are highlighted in blue.
(XLSX)

Text S1 **Primer sequences.**
(DOCX)
