# Supplementary figures and images for: Mediated Plastid RNA Editing in Plant Immunity
Source: PLoS Pathog. 2013 Oct 31;9(10):e1003713. doi: 10.1371/journal.ppat.1003713 (PMC3814343; doi:10.1371/journal.ppat.1003713)

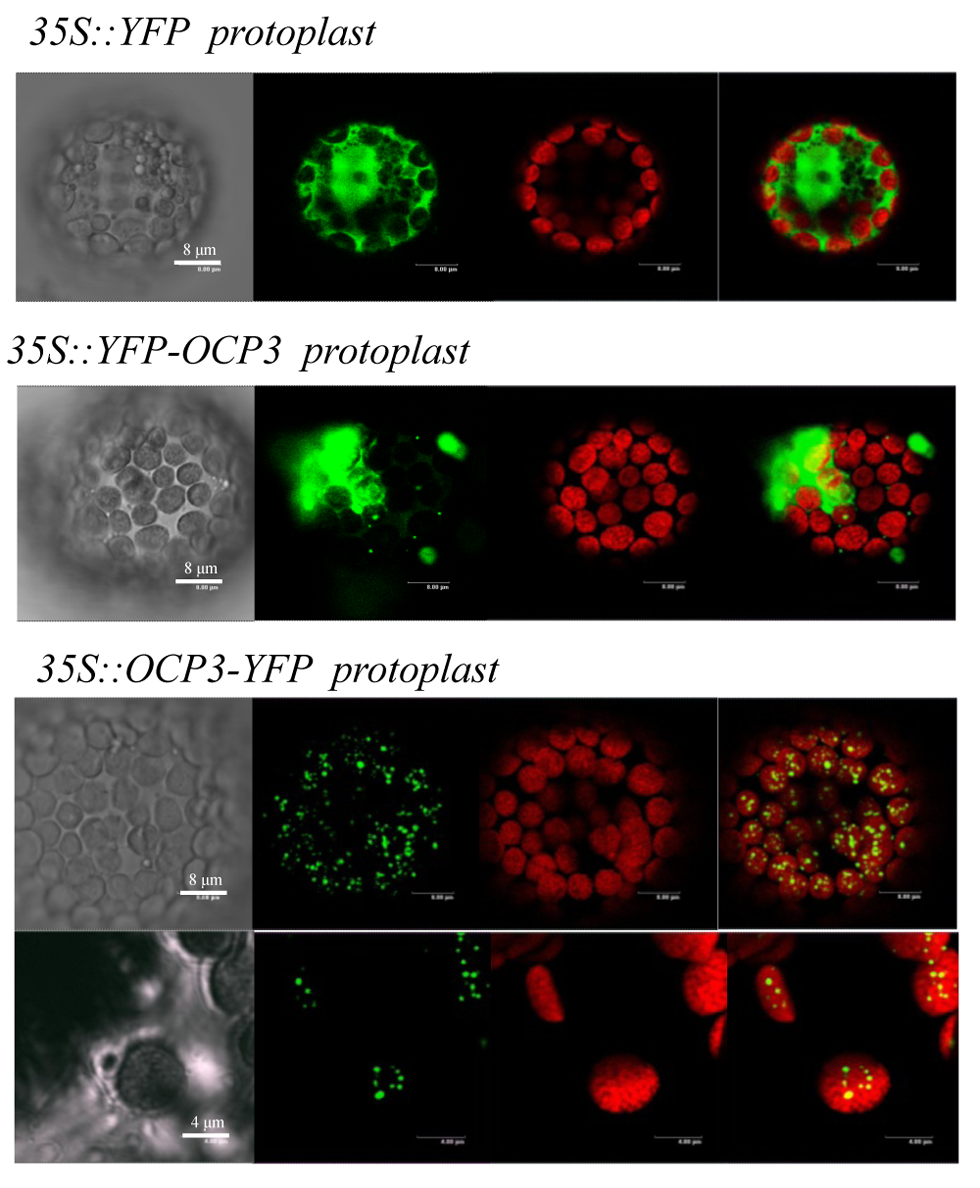

Supplement: Figure S1 — Protein localization in N. benthamiana protoplast. Fluorescent confocal microscopy evaluation of protein localization in transfected protoplast of N. benthamiana with a 35S::YFP construct (upper panel), a 35S::YFP-OCP3 construct (middle panel) and a 35S::OCP3-YPF constructs (lower panel). YFP-specific fluorescence is shown in green and chlorophyll-derived fluorescence is shown in red. (TIF) [file ppat.1003713.s001.tif]

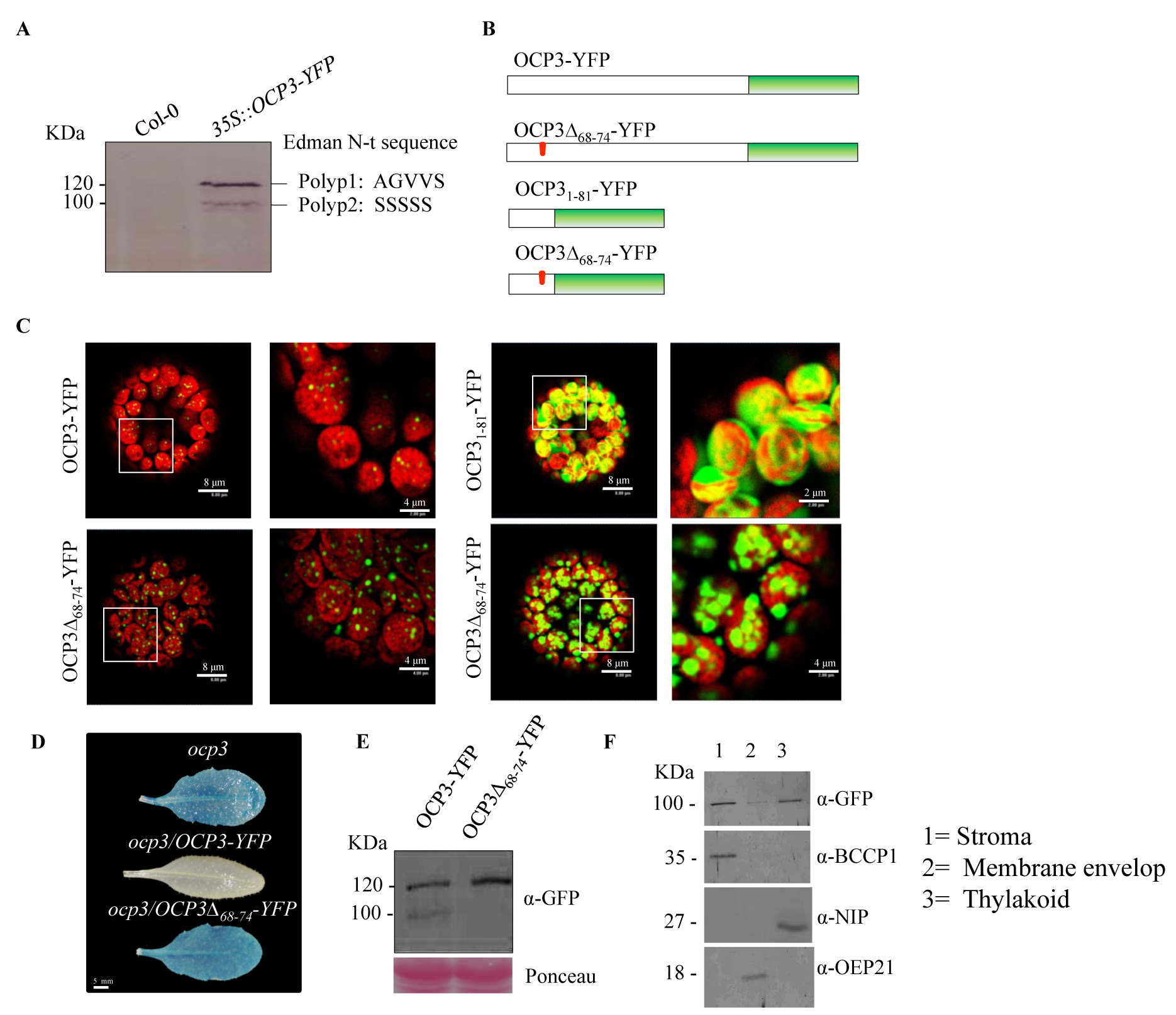

Supplement: Figure S2 — Functional characterization of the signal peptide sequence of OCP3. (A) SDS-PAGE and protein immunoblot with anti-GFP antibody indicating protein band position of OCP3-GFP precursors (Polyp1 and Polypep2) that were used for N-terminal amino acid sequence determination by Edman sequential degradation. Five rounds of degradation were conducted for each polypeptide which rendered the indicated 5 amino acid long N-terminal sequence. (B) Scheme depicting the different gene construct used for testing functionality of the OCP3 signal peptide. Green: YFP protein; white: OCP3 protein; red symbol: relative position where the Δ68–74 internal deletion in the signal peptide sequence was created. (C) Confocal microscopy localization of the relevant constructs shown in (B). (D) Comparative complementation test of the ocp3 mutant (which show constitutive GUS expression as driven by the Ep5C gene promoter) with construct 35S::OCP3-YFP and construct 35S::OCP368–74-YFP. The latter carries a deletion of 7 amino acids in the Signal peptide of OCP3 and was unable to be processed in the chloroplast. (E) Western blot with anti-GFP antibodies of proteins extracts derived from ocp3 plants transformed with 35S::OCP3-YFP and 35S::OCP368–74-YFP gene constructs. (F) Western blots of the indicated chloroplast compartments obtained from chloroplasts preparations derived from ocp3 plants transformed with the 35S::OCP3-YFP gene construct. Westerns were developed using anti-GFP; anti-BCCP1 (as marker for the stroma (lane 1; loaded with 4 µg total protein)); anti-OEP21 (as marker for membrane envelop (lane 2; loaded 1 µg total protein)); and anti-NIP (as marker for thylakoids (lane 3; loaded with 1 µg total protein). (TIF) [file ppat.1003713.s002.tif]

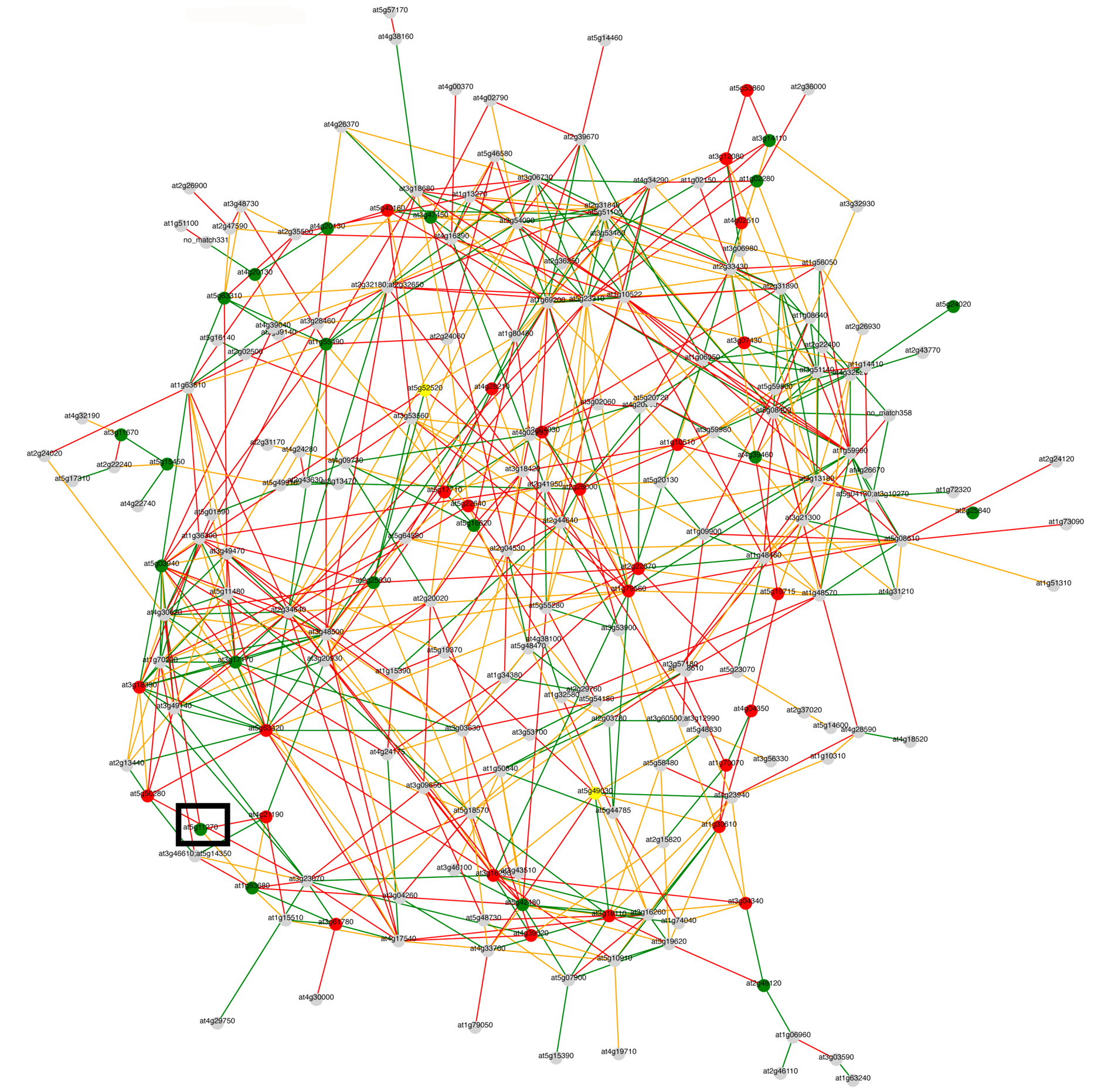

Supplement: Figure S3 — Co-expression gene vicinity network for OCP3. Nodes indicate individual genes, and edges indicate whether two genes are co-expressed above a certain mutual rank. Red, yellow, green, and grey nodes indicate whether mutations in the gene cause embryo lethality (red), gametophytic lethality (yellow), any other biological phenotype (green), or if no mutant phenotype currently is available (grey) according to TAIR. The color edges indicate strength of the coexpression based on mutual rank relationships between the individual gene pairs. Green, orange, and red edges indicate a mutual rank relationship ≤10 (green), between 11 and 20 (orange) and 21 and 30 (red), respectively, for each connected gene. The network was generated, and modified from AraGenNet (http://aranet.mpimp-golm.mpg.de/aranet; Mutwil et al., 2010). (TIF) [file ppat.1003713.s003.tif]

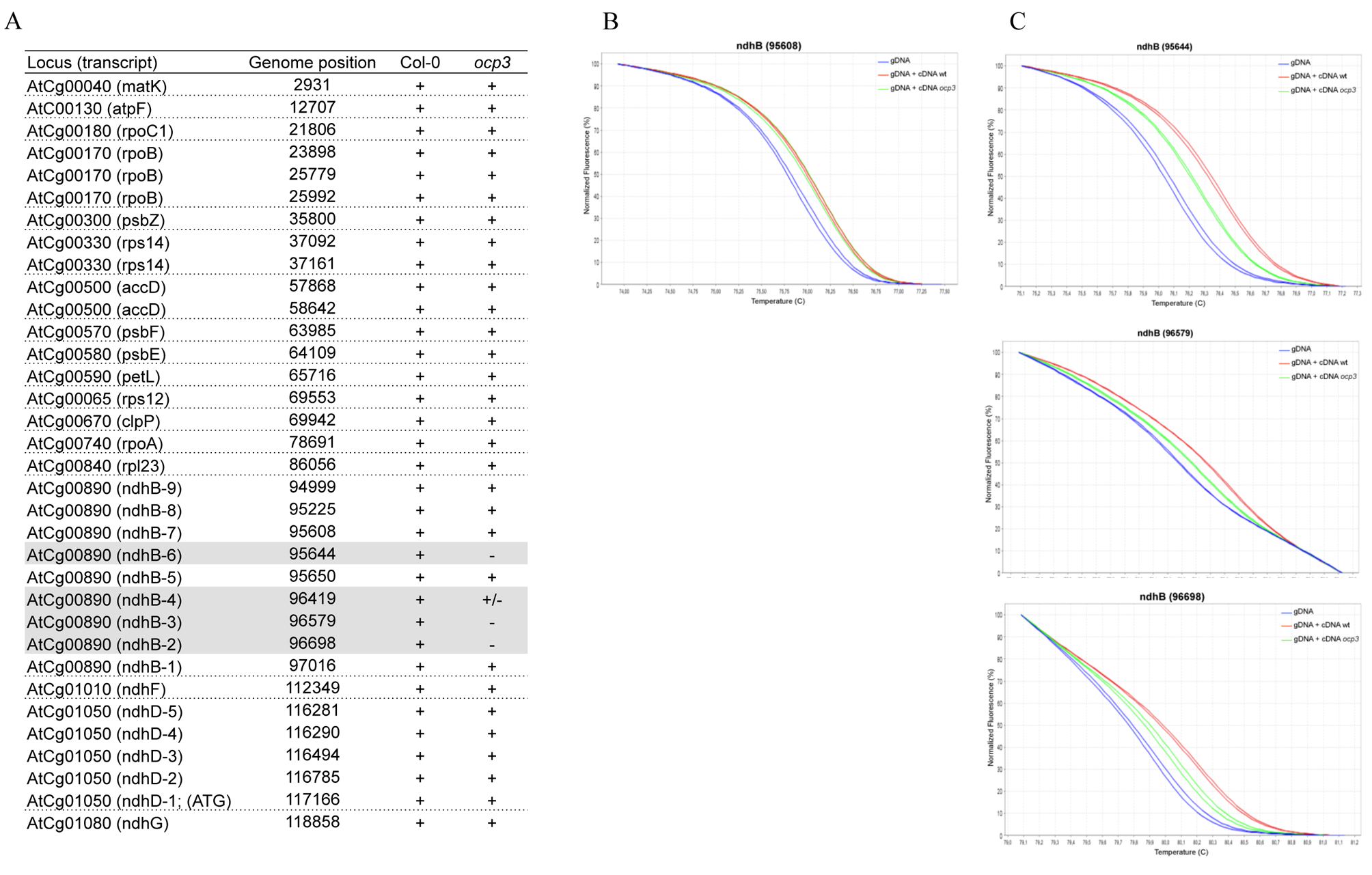

Supplement: Figure S4 — Analysis of the editotype of the ocp3 mutant as revealed by high resolution melting (HRM). (A) Editing of the current 34 sites in A. thaliana chloroplast transcripts. Editing regulated genes are shown in the first column. The exact positions in the chloroplast genome sequence of each edited nucleotide is shown in the next column. Observed changes by HRM between Col-0 and ocp3 plants are marked with (+) symbols, and absence of differences are marked with (−) symbol. The four editing sites found to be affected in ocp3 plants are dashed in grey. (B) Example of HRM analysis, monitored by decrease in fluorescence as the temperature increase, for the amplicon encompassing ndhB transcript at position 95608 (ndhB-7 site) which suffers no variation between Col-0 and ocp3. (C) Examples of HRM analysis where the presence of less thermostable heteroduplexes in a sample alters the shape of the melting curves such as occurs with amplicons for transcript ndhB at positions 95644 (dnhB-6 site), 96579 (ndhB-3 site) or 96698 (ndhB-2 site) which suffer variation between Col-0 and ocp3. (TIF) [file ppat.1003713.s004.tif]

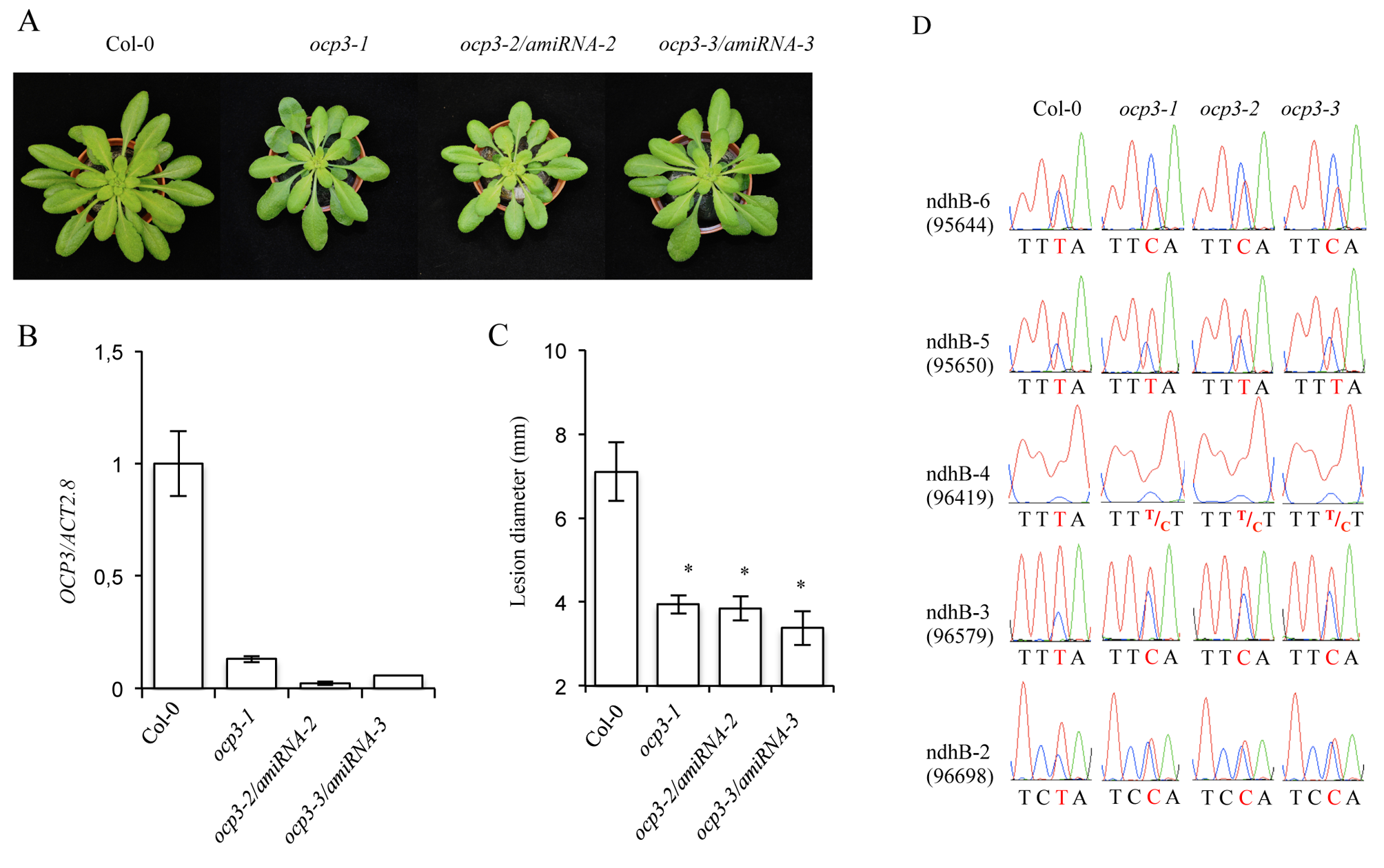

Supplement: Figure S5 — Characterization of Arabidospis strains silenced in OCP3 and generated artifitial microRNAs (amiRNAs). The new ocp3 mutant strains (ocp3-2/amiRNA-2 and ocp3-2/amiRNA-3) were generated by artifitial microRNAs (amiRNAs) designed to specifically target and down-regulate OCP3. The resulting mutant phenotypes were compared to the EMS-induced ocp3-1 mutant and Col-0. (A) Comparison of vegetative growth and anatomical appearance between Col-0, ocp3-1, ocp3-2 and ocp3-3. (B) RT-qPCR of OCP3 transcript levels in the four indicated genetic backgrounds. OCP3 expression was normalized to ACTIN2.8 expression. Bars represent mean ± SD, n = 3 independent replicates. (C) Lesion size resulting from P. cucumerina infection in Col-0, ocp3-1, ocp3-2, and ocp3-3 plants at 12 days post-inoculation. Values are means and ± SE (n = 50). Asterisks indicate significant differences (LSD test; P<0.05). (D) Sequence electrophoregrams corresponding to the RNA editing sites of ndhB-6 (95644), ndhB-5 (95650), ndhB-4 (96419), ndhB-3 (96579), ndhB-2 (96698) as derived from bulk RT-PCR sequencing of amplicons from Col-0, ocp3-1, ocp3-2, and ocp3-3 plants mRNA preparations. Editing sites are indicated by a red T residue and unedited sites by a red C residue. Partial editing inhibition is indicated by red T/C. (TIF) [file ppat.1003713.s005.tif]

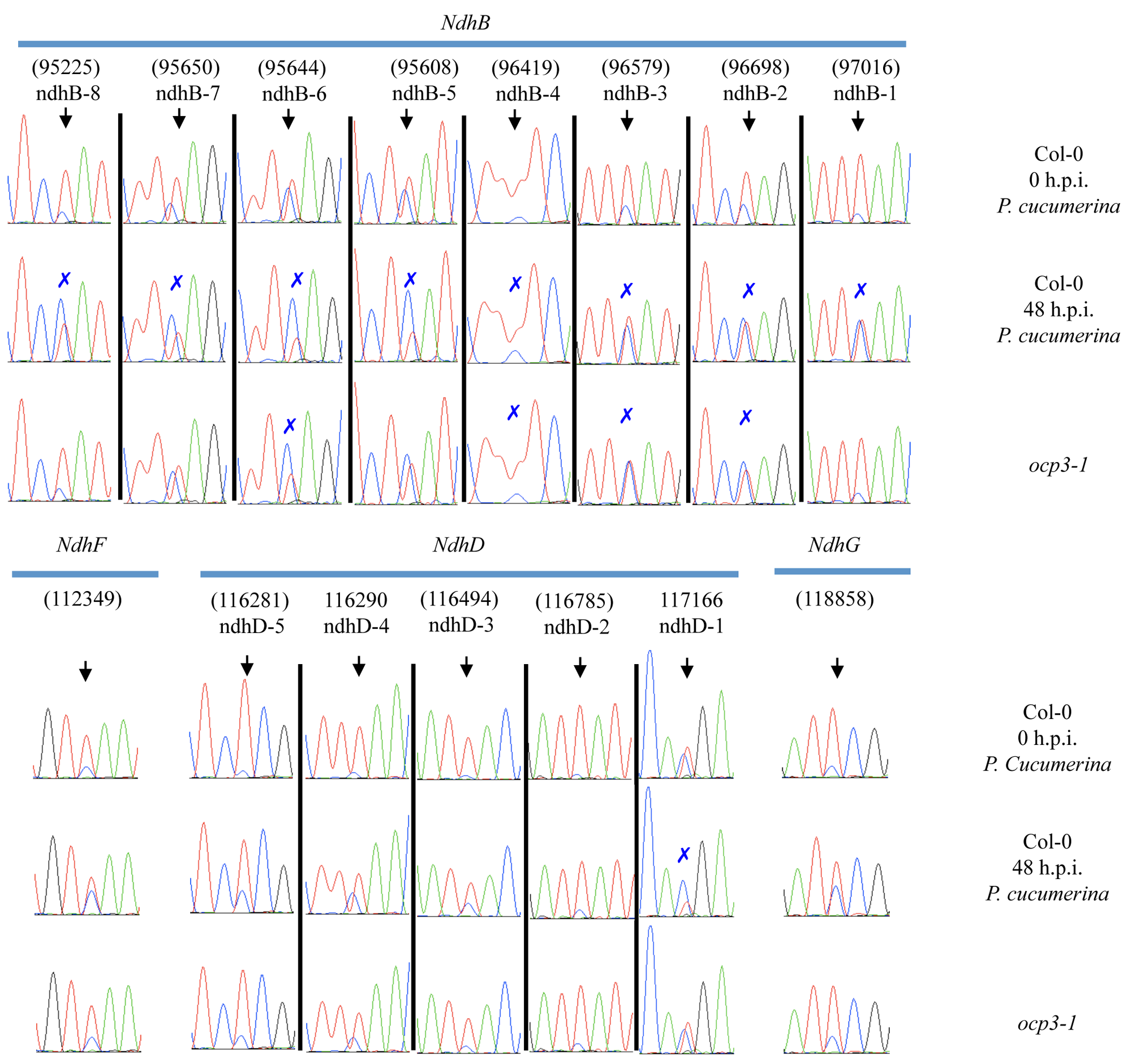

Supplement: Figure S6 — P. cucumerina -mediated editing inhibition. Nucleotide sequences of RT-PCR products obtained from Col-0 plants at 0 hours and at 48 hours post-inoculation with P. cucumerina, and its comparison with non-inoculated ocp3-1 plants, are shown as sequencing electrophoregrams. Editing sites for the four transcript encoding the chloroplast-encoded NDH complex subunits (i.e., NdhB, NdhD, NdhF, and NdhG) are indicated by arrows pointing to the corresponding peaks. Observed editing inhibition following P. cucumerina infection are marked with a blue cross above the corresponding editing site. (TIF) [file ppat.1003713.s006.tif]

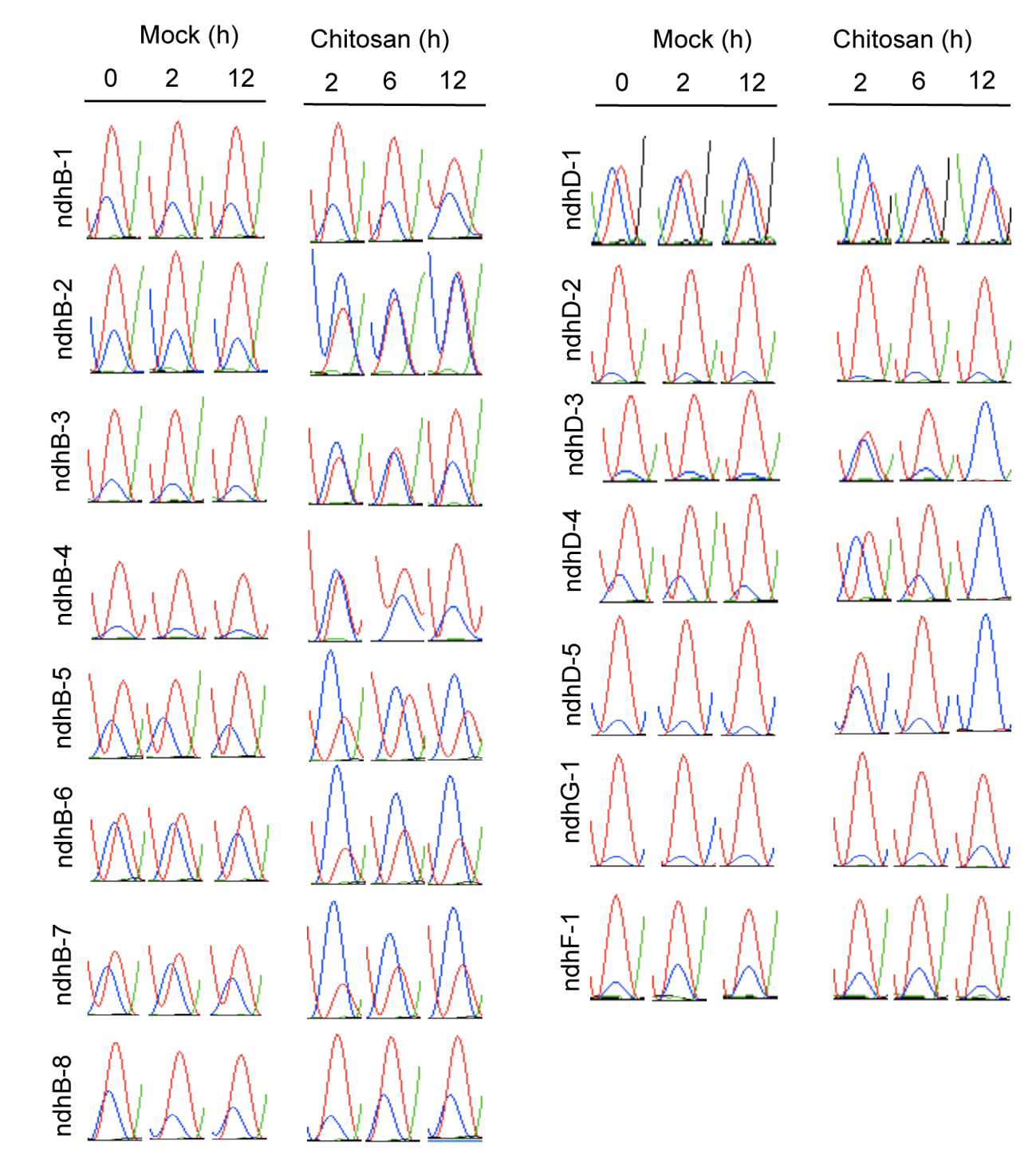

Supplement: Figure S7 — Sequencing electrophoregrams of nucleotide sequence of RT-PCR products obtained from Col-0 seedlings at the times indicated following mock or chitosan (10 µg/mL) treatment. The electrophoregrams show the C nucleotide either edited or not edited at the corresponding editing site of the corresponding transcript. Shown are editing sites for which chitosan exerts editing inhibition effect. (TIF) [file ppat.1003713.s007.tif]

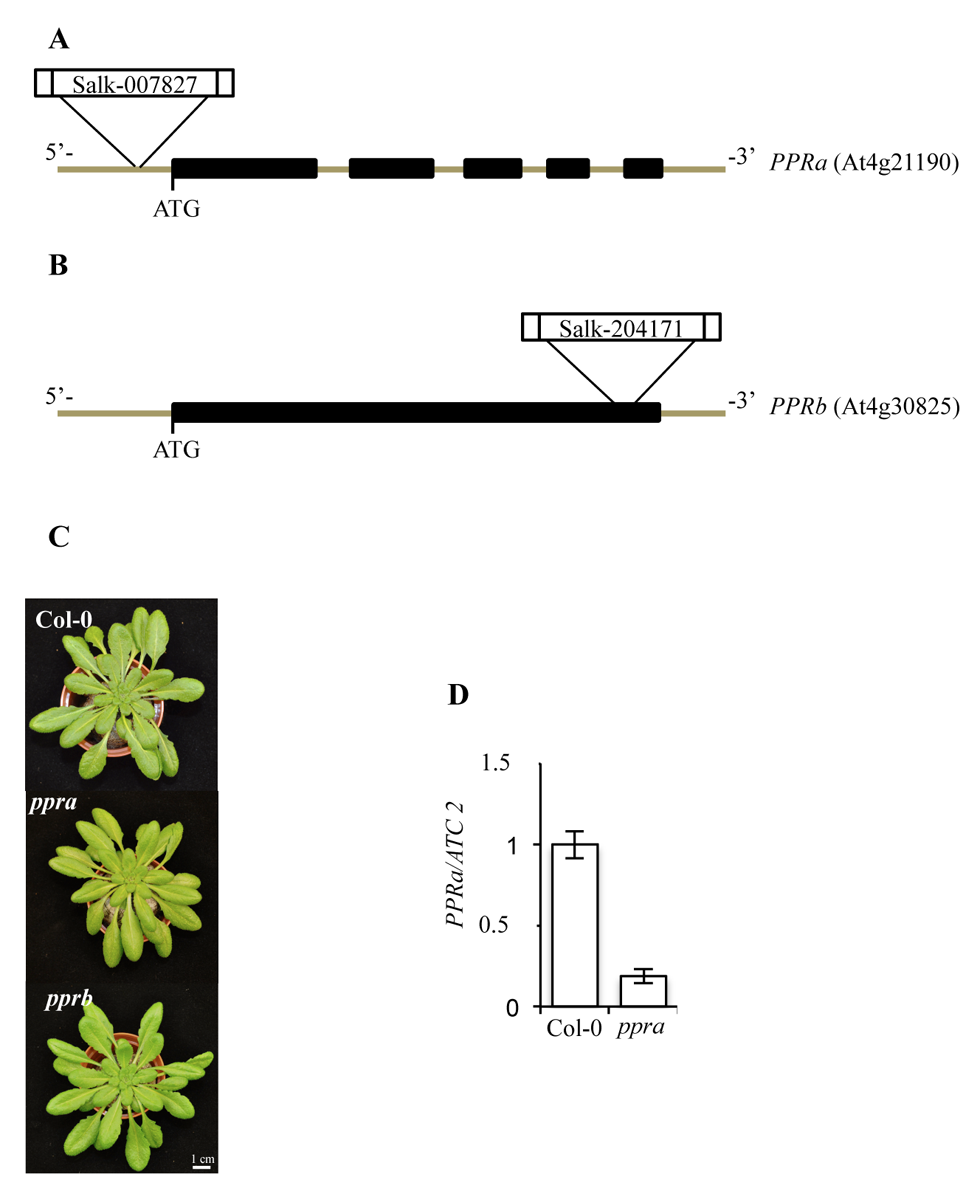

Supplement: Figure S8 — PPRa (At4g2119) and PPRb (At4g3082) T-DNA insertion mutants. (A) The ppra mutant (strain Salk-007827) carries a T-DNA insertion at 340 nt upstream of the ATG initiation codon and therefore could affect expression of the gene. (B) The pprb mutant (strain Salk-204171) carries a T-DNA insertion internal to the unique exon, close to the ATG initiation codon, and therefore disrupts the ORF. Exons are indicated with solid rectangles. T-DNA insertions are indicated with white rectangles. (C) None of the mutations affect the normal growth of the plants and both mutants resemble Col-0 plants in morphological phenotype. (D) RT-qPCR of PPRa transcript levels in Col-0 and in ppra mutant reveal that expression of PPRa was down-regulated in the mutant. PPRa expression was normalized to ACTIN2.8 expression. Bars represent mean ± SD, n = 3 independent replicates. (TIF) [file ppat.1003713.s008.tif]
