# Supplementary material for: Mediated Plastid RNA Editing in Plant Immunity
Source: PLoS Pathog. 2013 Oct 31;9(10):e1003713. doi: 10.1371/journal.ppat.1003713 (PMC3814343; doi:10.1371/journal.ppat.1003713)
Supplement: Text S1 — Primer sequences. (DOCX) [file ppat.1003713.s011.docx]

**Text S1. Primer sequences.**

*Relationship of primers used in this study.*

| Primer name | Nucleotide sequence (5` to 3`) | Use |
| --- | --- | --- |
| OCP3-BP-Fw | ggggacaagtttgtacaaaaaagcaggctat*atg*atgataaaagccatggctctatcttc | Cloning OCP3 in pDonr221 |
| OCP3-BP-Rv-STOP | ggggaccactttgtacaagaaagctgggta*tta*tcaaaccggagcttgatatgg | Cloning OCP3 in pDonr221 |
| OCP3-BP-Rv-NO STOP | ggggaccactttgtacaagaaagctgggta*taa*tccaaccggagcttgatatgg | Cloning OCP3 in pDonr221 |
| PGL34-BP-FW | ggggacaagtttgtacaaaaaagcaggctat*atg*gcattgatccaacatg | Cloning PGL34 in pDonr221 |
| PGL34-BP-Rv-NO STOP | ggggaccactttgtacaagaaagctgggta*taa*actgttgtattcaagattctctacaac | Cloning PGL34 in pDonr221 |
| PTAC2-BP-Fw | ggggacaagtttgtacaaaaaagcaggctat*atg*aaacctagcaattcc | Cloning pTAC2 in pDonr221 |
| PTAC2-BP-Rv-NO STOP | ggggaccactttgtacaagaaagctgggta*taa*agctgtgctccctgc | Cloning pTAC2 in pDonr221 |
| Δ_68-74_OCP3-Fw | ccgaagaaaaacaaaaagaaaagtttgg | Introduce mutation in OCP SP |
| Δ_68-74_OCP3-Rv | gaatccttttcgattcttgccac | Introduce mutation in OCP SP |
| Nt-OCP3-BP-Fw | ggggacaagtttgtacaaaaaagcaggctat*atg*ataaaagccatggctc | Cloning SP sequence in pDonr221 |
| Nt-OCP3-BP-Rv | ggggaccactttgtacaagaaagctgggtatttctttttgtttttcttcg | Cloning SP sequence in pDonr221 |
| PPRa-BP-Fw | ggggacaagtttgtacaaaaaagcaggctat*atg*ctaagcttgag | Cloning PPRa pDonr207 |
| PPRa-BP-Rv-NO STOP | ggggaccactttgtacaagaaagctgggtactttgaagagtcc | Cloning PPRa pDonr207 |
| CRR21-BP-Fw | ggggacaagtttgtacaaaaaagcaggctat*atg*gcttctcttcctttc | Cloning CRR21 in pDonr207 |
| CRR21-BP-Rv-No STOP | ggggaccactttgtacaagaaagctgggtactttttggaacctgtaccca | Cloning CRR21 in pDonr207 |
| CRR2-BP-Fw | ggggacaagtttgtacaaaaaagcaggctat*atg*tttctgtctcaccc | Cloning CRR2 in pDonr207 |
| CRR2-BP-Rv-NO STOP | ggggaccactttgtacaagaaagctgggtacttccagtaatctccac | Cloning CRR2 in pDonr207 |
| PPRb-BP-Fw | Ggggacaagtttgtacaaaaaagcaggctat*atg*ggttcgttaagg | Cloning PPRb in pDonr207 |
| PPRb-BP-Rv-NO STOP | ggggaccactttgtacaagaaagctgggtactttatacccatctgtttca | Cloning PPRb in pDonr207 |

| PPE ndhB-4 | tcgaaagtagctgcttcagc | PPE assay |
| --- | --- | --- |
| PPE ndhB-5 | gttacttcgaaagtagctgc | PPE assay |
| PPE ndhB-6 | ttcaagctttccctagcccc | PPE assay |
| PPE ndhB-7 | tggggcaagctcttctattc | PPE assay |

| PPRa KO-FW | agcttgtttgcaaaggcttac | *ppra* T-DNA mutant genotyping |
| --- | --- | --- |
| PPRa KO-Rv | cgaaccacaacattgttaggc | *ppra* T-DNA mutant genotyping |
| PPRb KO-Fw | cagcagaaagacattgttccg | *pprb* T-DNA mutant genotyping |
| PPRb KO-Rv | ctaatccacgtcatccgaaac | *pprb* T-DNA mutant genotyping |

| qRT-PPRa-FW | caggtgacaaaatggatgctgag | RT-qPCR |
| --- | --- | --- |
| qRT-PPRa-Rv | agcttcgtcaaggcgattatcttc | RT-qPCR |
| qRT-CRR21-Fw | tgcagtgaggaggcaattctg | RT-qPCR |
| qRT-CRR21-Rv | atgctagatgtgcgcaagcag | RT-qPCR |
| qRT-PPRb-Fw | gtttgttccaccgcctctgta | RT-qPCR |
| qRT-PPRb-Rv | tgcctcctcgtaattgtcagc | RT-qPCR |
| qRT-OCP3-Fw | aagctgggcgtcgtaaaactagta | RT-qPCR |
| qRT-OCP3-Rv | tggcggtttttcatctggtagtgt | RT-qPCR |
| qRT-PDF1.2a-Fw | cttgttctctttgctgctttc | RT-qPCR |
| qRT-PDF1.2a-Rv | catgtttggctccttcaag | RT-qPCR |
| qRT-ACT2-Fw | tcttccgctctttctttccaagc | RT-qPCR |
| qRT-ACT2-Rv | accattgtcacacacgattggtt | RT-qPCR |

| CHLORO 27 Fw | cagcctattcttgaaatgaacaac | HRM atpF (12707 position) |
| --- | --- | --- |
| CHLORO 27 Rv | gattcgctttcttccttccc | HRM atpF (12707 position) |
| CHLORO 45 FW | aaatttcagggtagcaaacattc | HRM rpoB (25779;26992 position) |
| CHLORO 45 RV | aaattgaagatatagatcacgaaattga | HRM rpoB (25779;26992 position) |
| CHLORO 68 FW | agaacatagccctatgagttaatacga | HRM psbZ (35800 position) |
| CHLORO 68 RV | gataagagaattaaggatacccacca | HRM psbZ (35800 position) |
| CHLORO 72 FW | tccatttctcacttagcgacg | HRM rps14 (27161 position) |
| CHLORO 72 RV | ttcttaatttcctggcgtgg | HRM rps14 (27161 position) |
| CHLORO 112 FW | aaattacaagataagaactaataggaatcg | HRM accD (57868 position) |
| CHLORO 112 RV | agaggtaaacattgattggtagcat | HRM accD (57868 position) |
| CHLORO 113 FW | tggatgcaattgtaccacgta | HRM accD (58642 position) |
| CHLORO 113 RV | agaatctgatctaacaacagggaa | HRM accD (58642 position) |
| CHLORO 125 FW | acgtctataactcagctcatccaa | HRM psbF (63985 position) |
| CHLORO 125 RV | catggactagctgtacctaccgt | HRM psbF (63985 position) |
| CHLORO 130 FW | gccaaagacgatgaattgagtt | HRM petL (65716 position) |
| CHLORO 130 RV | gagaggtactatctattcctagctcttaatc | HRM petL (65716 position) |
| CHLORO 140 FW | cttgtacaattcacattctttggc | HRM rps12 (69553 position) |
| CHLORO 140 RV | caagacagccaatccgaaac | HRM rps12(69553 position) |
| CHLORO 141 FW | acatgttcctcgtcgctga | HRM clpP (69942 position) |
| CHLORO 141 RV | tttatgaggcacaaacggga | HRM clpP (69942 position) |
| CHLORO 159 FW | ggagttaaacttccatttgtcca | HRM rpoA (78691 position) |
| CHLORO 159 RV | gcgatgcgaagagctttact | HRM rpoA (78691 position) |
| CHLORO 178 FW | aagaggtggaatagaataacccg | HRM rpl23 (86056 position) |
| CHLORO 178 RV | aattcctactggatgcacgc | HRM rpl23 (86056 position) |
| CHLORO 185 FW | aggtcagatttcgcctattcc | HRM ndhB (94999 position) |
| CHLORO 185 RV | tgactggacgaaaccaagaa | HRM ndhB (94999 position) |
| CHLORO 186 FW | ttctataatttcgcatgtgaggg | HRM ndhB (95225 position) |
| CHLORO 186 RV | tcattgctattactcaaacaagca | HRM ndhB (95225 position) |
| CHLORO 187 FW | tccgatttgacctatggacg | HRM ndhB (95608;95644;95650 positions) |
| CHLORO 187 RV | gtgctcctacggaaccaagg | HRM ndhB (95608;95644;95650 positions) |
| CHLORO 189 FW | tctcagagatagatagagaggtaggaattt | HRM ndhB (96419;96579;96698 positions) |
| CHLORO 189 RV | cattgaatgtacagaaatggctataa | HRM ndhB (96419;96579;96698 positions) |
| CHLORO 190 FW | aaacattcctcctagagtagctgttaat | HRM ndhB (97016 position) |
| CHLORO 190 RV | ttccgtaaatattccattccaaa | HRM ndhB (97016 position) |
| CHLORO 204 FW | ggtatccttgatcatgcgacata | HRM ndhF (112349 position) |
| CHLORO 204 RV | tcaatattcatggatcatcccttt | HRM ndhF (112349position) |
| CHLORO 212 FW | agcgccaataaatccatgag | HRM ndhD (116281;116290;116494 positions) |
| CHLORO 212 RV | gttcgaacgaaccaacattaaa | HRM ndhD (116281;116290;116494 positions) |
| CHLORO 213 FW | aatagtatttcaagtgtaacaggatagga | HRM ndhD (116785 positions) |
| CHLORO 213 RV | tggacgatccattaattcaact | HRM ndhD (116785 positions) |
| CHLORO 214 FW | tcgatccatttataatcttcgga | HRM ndhD (117166 positions) |
| CHLORO 214 RV | gactgtgttggttgtaagagatgtg | HRM ndhD (117166 positions) |
| CHLORO 218 FW | cttattaaatcttgctctagaatctggtt | HRM ndhG (118858 positions) |
| CHLORO 218 RV | ataatggatttgcctggacc | HRM ndhG (118858 positions) |
| CHLORO 260 FW | aagatgtcgatcgtaaatgcg | HRM matK (2931 position) |
| CHLORO 260 RV | tcgtttgattattcccactaagg | HRM matK (2931 position) |
| CHLORO 39 FW | ttcgcaaatctaaatcggct | HRM rpoC1(21806position) |
| 21861 RV | ttttcttttgctaggcccataa | HRM rpoC1(21806position) |
| 23581 FW | atggattcgctgtttgcttactg | HRM rpoB (23898 position) |
| 24093 RV | ctcaagtggcgaaagaatcc | HRM rpoB (23898 position) |
| RPS14.AT.Rv | taccagcttgatcttgttgc | HRM rps14 (37092position) |
| CHLORO 71RV | tcatttgattcgtcgatcctc | HRM rps14 (37092position) |
